# The MHV-68 nuclear egress complex supports C-capsid selective capsid egress

**DOI:** 10.1128/jvi.00072-26

**Published:** 2026-04-17

**Authors:** Saskia Sanders, Carola Schneider, Timothy K. Soh, Elena Kotova, Dörte Stalling, Beatrix Steer, Rudolph Reimer, Zsolt Ruzsics, Heiko Adler, Jens B. Bosse

**Affiliations:** 1 Hannover Medical School, Institute of Virology 686461, Hanover, Germany; 2 Centre for Structural Systems Biology, Hamburg, Germany; 3 Cluster of Excellence RESIST (EXC 2155), Hannover Medical School 9177, Hanover, Germany; 4 Leibniz Institute of Virology (LIV) 28367, Hamburg, Germany; 5 Institute of Asthma and Allergy Prevention, Helmholtz Zentrum München, German Research Center for Environmental Health, Neuherberg, Germany; 6 German Center for Lung Research (DZL/CPC-M), Munich, Germany; 7 University Medical Center Freiburg, Institute of Virology 14879, Freiburg, Germany; 8 Walther Straub Institute of Pharmacology and Toxicology, Ludwig-Maximilians-University Munich 9183 , Munich, Germany; Northwestern University Feinberg School of Medicine, Chicago, Illinois, USA

**Keywords:** nuclear egress complex, NEC, murine gammaherpesvirus 68, MHV-68, herpesvirus

## Abstract

Herpesvirus capsids assemble within the host nucleus and must traverse the nuclear envelope to undergo final envelopment in the cytoplasm. This exit strategy is orchestrated by the nuclear egress complex (NEC), a conserved heterodimeric protein complex that forms lattices that remodel the inner nuclear membrane. While the NEC is critical for efficient replication across α-, β-, and γ-herpesviruses, residual infectivity has been noted in NEC-deficient mutants of the ɑ-herpesvirus Pseudorabies virus. Here, we investigated the NEC’s role in the γ-herpesvirus murine γ-herpesvirus 68 (MHV-68) by generating a mutant virus (ΔC-ORF69) lacking the majority of the nucleoplasmic NEC component, ORF69. Unlike prior studies utilizing transfection-based systems, we demonstrate that NEC-deficient MHV-68 produces infectious progeny in non-complementing cells, albeit with a significant replication defect. Quantitative electron microscopy confirmed that while nuclear capsid assembly remained regular, the canonical export mechanism was abolished. Whereas the parental virus predominantly exported mature, enveloped, genome-filled C-capsids out of the nucleus, the ΔC-ORF69 mutant released all major capsid forms (A, B, and C) into the cytoplasm and extracellular space, associated with loss of nuclear integrity. These findings suggest that, in addition to mediating efficient capsid translocation across the nuclear envelope, the MHV-68 NEC imposes C-capsid selectivity during nuclear egress, thereby acting as a quality control checkpoint. Inhibition of cyclin-dependent kinases reduced viral spread in both parental and ΔC-ORF69 viruses, indicating that cyclin-dependent kinase activity contributes to productive infection and may influence the residual spread observed in ΔC-ORF69, consistent with a potential contribution to late-stage nuclear envelope integrity loss.

**IMPORTANCE:**

Human γ-herpesviruses, such as Epstein-Barr virus and Kaposi’s sarcoma-associated herpesvirus, are significant pathogens that cause lifelong infections and malignancies. A critical bottleneck in their life cycle is the translocation of viral capsids from the nucleus to the cytoplasm, a process driven by the viral nuclear egress complex (NEC). By characterizing a specific NEC mutant in the murine model MHV-68, we uncovered a fundamental, previously underappreciated function of this complex. While the NEC is often primarily viewed as transport machinery, our data demonstrate that it functions as a critical specificity determinant, selectively allowing only mature, DNA-filled capsids to exit the nucleus. In the absence of a functional NEC, this selectivity is lost, leading to the non-selective leakage of all major capsid forms via nuclear envelope disruption.

## INTRODUCTION

The Gammaherpesvirinae subfamily includes profound human pathogens such as Epstein-Barr virus (EBV) and Kaposi’s sarcoma-associated herpesvirus (KSHV), which are causative agents of various malignancies (1). A defining characteristic of herpesvirus replication is the spatial separation of viral assembly: DNA replication and capsid formation occur in the host nucleus, while final maturation occurs in the cytoplasm. Because the murine γ-herpesvirus 68 (MHV-68) undergoes robust lytic replication in culture, unlike the predominantly latent EBV and KSHV, it serves as a valuable γ-herpesviral model for dissecting conserved mechanisms like the nuclear-cytoplasmic translocation of capsids (1, 2). The synthesis of viral progeny in the nucleus yields three distinct major capsid forms: A-capsids (empty capsids), B-capsids (containing protein scaffolds but no DNA), and C-capsids (containing the viral dsDNA genome) (3, 4). For efficient progeny production, the virus must export the genome-filled C-capsids through the nuclear envelope for further maturation in the cytoplasm. This translocation occurs via a vesicle-mediated process termed nuclear egress, where capsids bud through the inner nuclear membrane into the perinuclear space (primary envelopment) and subsequently fuse with the outer nuclear membrane to release the capsid into the cytoplasm (1). This distinct membrane remodeling event is driven by the viral nuclear egress complex (NEC). The NEC is a conserved heterodimer consisting of a membrane-anchored protein (ORF67 in MHV-68) and a soluble nucleoplasmic partner (ORF69 in MHV-68) (5). Structural studies in α- and β-herpesviruses have revealed that homologs of these proteins interlock via a specific hook-into-groove interaction (6–10). Once assembled, these heterodimers oligomerize into hexagonal lattices that deform the nuclear membrane around the viral capsid (11–14). However, substantial gaps remain in our understanding of γ-herpesvirus egress. Specifically, it is unclear whether the NEC acts merely by transporting capsids or if it actively functions as a quality control checkpoint to prevent the release of defective A- and B-capsids. Furthermore, the strict essentiality of the NEC is debated. While deletion of NEC components in ɑ-herpesviruses generally blocks efficient nuclear egress (15–17), residual infectivity based on nuclear envelope breakdown (NEBD) has been observed (18–20), and dilated nuclear pores (21) have been proposed as alternative mechanisms of nuclear egress. In this study, we dissect the mechanism of MHV-68 nuclear egress by characterizing a targeted mutant of the nucleoplasmic NEC component, ORF69. Guided by AlphaFold predictions, we engineered a C-terminally truncated frameshift mutant (ΔC-ORF69) that retains the conserved N-terminal hook, which contains the amino acids required for the interaction with its membrane partner ORF67 (5), but lacks the C-terminal domain and is predicted to have lost its ability to oligomerize. By challenging this mutant in a full infection model, we demonstrate that while the wild-type NEC exports mostly mature C-capsids, the disruption of the ORF69 C-terminus results in the low-level leakage of all major capsid forms (A, B, and C) late in infection. Our data redefine the γ-herpesvirus NEC as a critical specificity determinant and reveal that in its absence, the virus may partially exploit host CDK-dependent NEBD events, resulting in a non-selective, low-level release of capsids.

## RESULTS

### Design of MHV-68 ΔC-ORF69

To dissect the mechanistic role of the NEC in the lytic infection of γ-herpesviruses, we required a system that allows for the monitoring of capsid dynamics in living cells. Although several reporter viruses exist for MHV-68 (22–24), no virus mutants with fluorescently tagged NECs have been reported, which limits direct visualization of nuclear egress. To enable tracking of capsid localization as a proxy for nuclear egress events, we utilized a previously characterized MHV-68 mutant expressing mCherry-fused small capsid protein ORF65 (25). This mutant virus is referred to hereafter as the parental virus.

To define the NEC’s role, we sought to decouple the requirement of heterodimer formation from the downstream processes of lattice assembly and capsid capture. A traditional knockout might fail to re-localize ORF67 to the nuclear rim and to distinguish between these steps, as ORF69 interaction with ORF67 is needed for correct re-localization of ORF67 (5). Therefore, we pursued a separation-of-function strategy designed to preserve the NEC core while selectively ablating its effector functions. Previous studies have shown that ORF69 and its homologs are required for proper nuclear rim localization of their membrane partners across herpesviruses (5, 16, 17, 26, 27). Specifically, the N-terminal hook domain of ORF69 contains a conserved cluster of residues, F52, F65, L66, and E68, which has been identified as critical for the ORF67-ORF69 interaction, as alanine substitution of these residues disrupts the interaction (5). Furthermore, this N-terminal region contains highly basic clusters, which in other herpesviruses have been associated with accessory functions such as DNA replication or membrane budding (28, 29).

**Fig 1:**
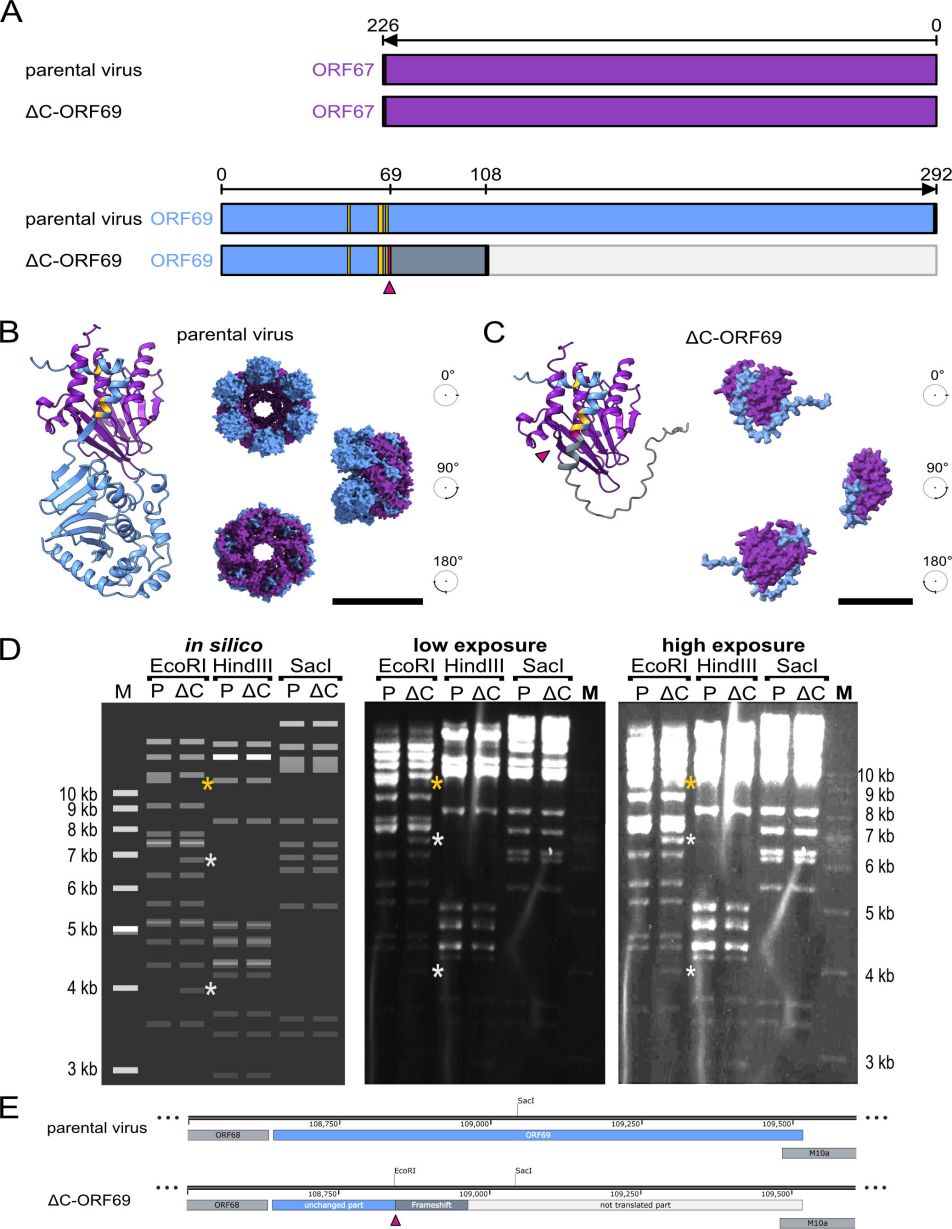
Design of the MHV-68 ΔC-ORF69 mutant. (**A**) Schematic of ORF67 and ORF69 in the parental virus and ΔC-ORF69. ΔC-ORF69 was generated by mutating the third in-frame ATG of ORF69 (codon 69, pink arrowhead), introducing a frameshift and premature stop at codon 108 (thick black line). ORF67 is shown in purple, the native ORF69 region in blue, the frameshifted region in blue-gray, and the non-expressed downstream region in light gray. Residues mediating ORF69-ORF67 interaction (E52, F65, L66, E68) are highlighted in gold. (**B**) AlphaFold 3 prediction of the ORF67-ORF69 heterodimer (ipTM = 0.75, pTM = 0.72) and a hexameric complex, shown as three surface views rotated by 90°. Known interface residues E52, F65, L66, and E68 are highlighted in gold. The first 42 N-terminal residues of ORF69 and the C-terminal 34 residues of ORF67, predicted to be disordered, are omitted for clarity. Scale bar: 100 Å. (**C**) AlphaFold 3 prediction of the ORF67-ΔC-ORF69 heterodimer (ipTM = 0.74, pTM = 0.65), with three surface views rotated by 90°. Interface residues are highlighted in gold as in panel B, and the mutated codon 69 is indicated in pink (triangle). Disordered N- and C-terminal segments are omitted as in panel B. Scale bar: 50 Å. (**D**) BACs of the parental virus and ΔC-ORF69 were digested with EcoRI, HindIII, and SacI and left, *in silico* digests for parental virus (P) and ΔC-ORF69 (ΔC); middle, low-exposure gel image; right, high-exposure image. Asterisks mark fragments altered in the EcoRI digest: fragment present only in the parental virus (gold asterisk); two smaller fragments in ΔC-ORF69 generated by the additional EcoRI site (white asterisks). (**E**) Schematic of the ORF69 locus in the parental virus and ΔC-ORF69. The codon 69 mutation (pink triangle) creates the additional EcoRI site.

The C-terminus of ORF69 homologs contains domains responsible for oligomerization, lattice formation (11, 29, 30), and capsid interaction (31). Therefore, we designed the ΔC-ORF69 mutant predicted to retain the structural integrity of the hook-into-groove interaction while ablating its higher-order functions. Using BAC mutagenesis, we altered the third in-frame ATG at codon 69 (substituting A for T and removing the subsequent TG). This mutation introduces a frameshift starting at position 69 and a premature stop codon at position 108 (Fig. 1A and E). Crucially, this strategy preserves the native amino acid sequence from residues 1 to 68, encompassing the basic clusters and all four critical interaction residues, while deleting the C-terminal domains. This design introduces a diagnostic EcoRI site (5′-GAATTC-3′) for screening purposes. BAC digestion using EcoRI, HindIII, and SacI confirmed the correct mutagenesis compared to the parental virus (Fig. 1D and E). This confirmed the generation of a mutant that was predicted to interact with ORF67, but was unlikely to oligomerize into a complex, unlike the parental virus (Fig. 1B and C). However, these predictions do not experimentally confirm whether the truncated protein retains the function to be recruited in infected cells.

**Fig 2:**
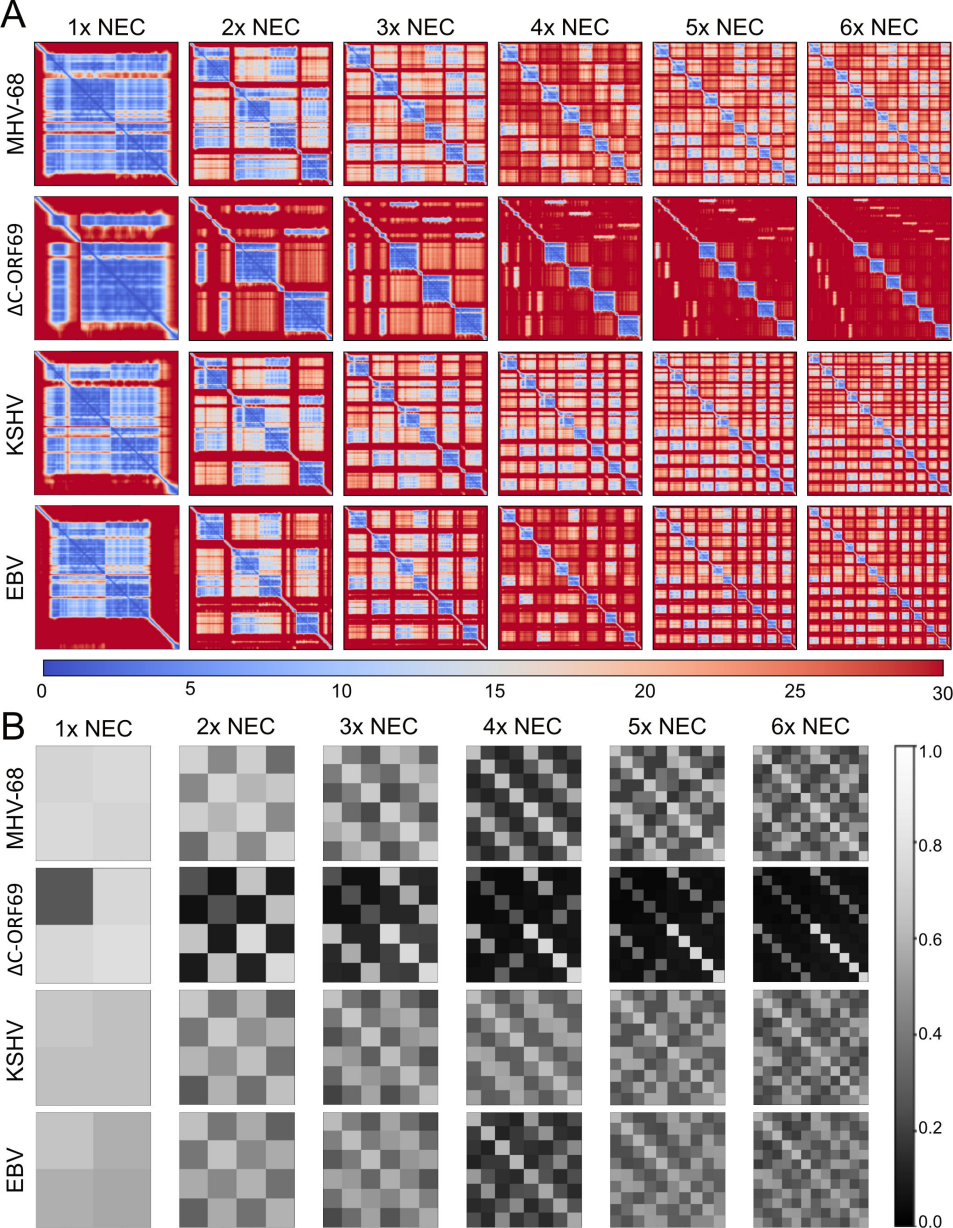
Quality assessment of AlphaFold 3 predictions of γ-herpesviruses of NEC oligomers. Visualization of different AlphaFold 3 quality scores for NEC-heterodimer oligomers (heterodimer to heterododecamer) in MHV-68, ΔC-ORF69, KSHV, and EBV. (**A**) PAE plots for the NEC oligomers. The heatmap represents the expected distance error in Å between residue pairs, with colors ranging from red (high error: 30 Å) to blue (low error: 0 Å); diagonal blocks reflect intra-chain confidence, and off-diagonal blocks reflect inter-chain placement confidence. For legibility, residue numbers are omitted; axes represent concatenated chain copies in AlphaFold 3 input order, with all ORF69-family chains (and homologs) followed by all ORF67-family chains (and homologs), yielding block-wise chain-chain regions. (**B**) Pairwise inter-chain ipTM confidence shown as a grayscale matrix. The scale ranges from black (low confidence: 0) to white (high confidence: 1), using the same order as in panel A. The corresponding PAE plots, including axis labels, pairwise ipTM comparisons, and pLDDT scores for each virus and oligomer, can be accessed at https://zenodo.org/records/15147695.

**Fig 3:**
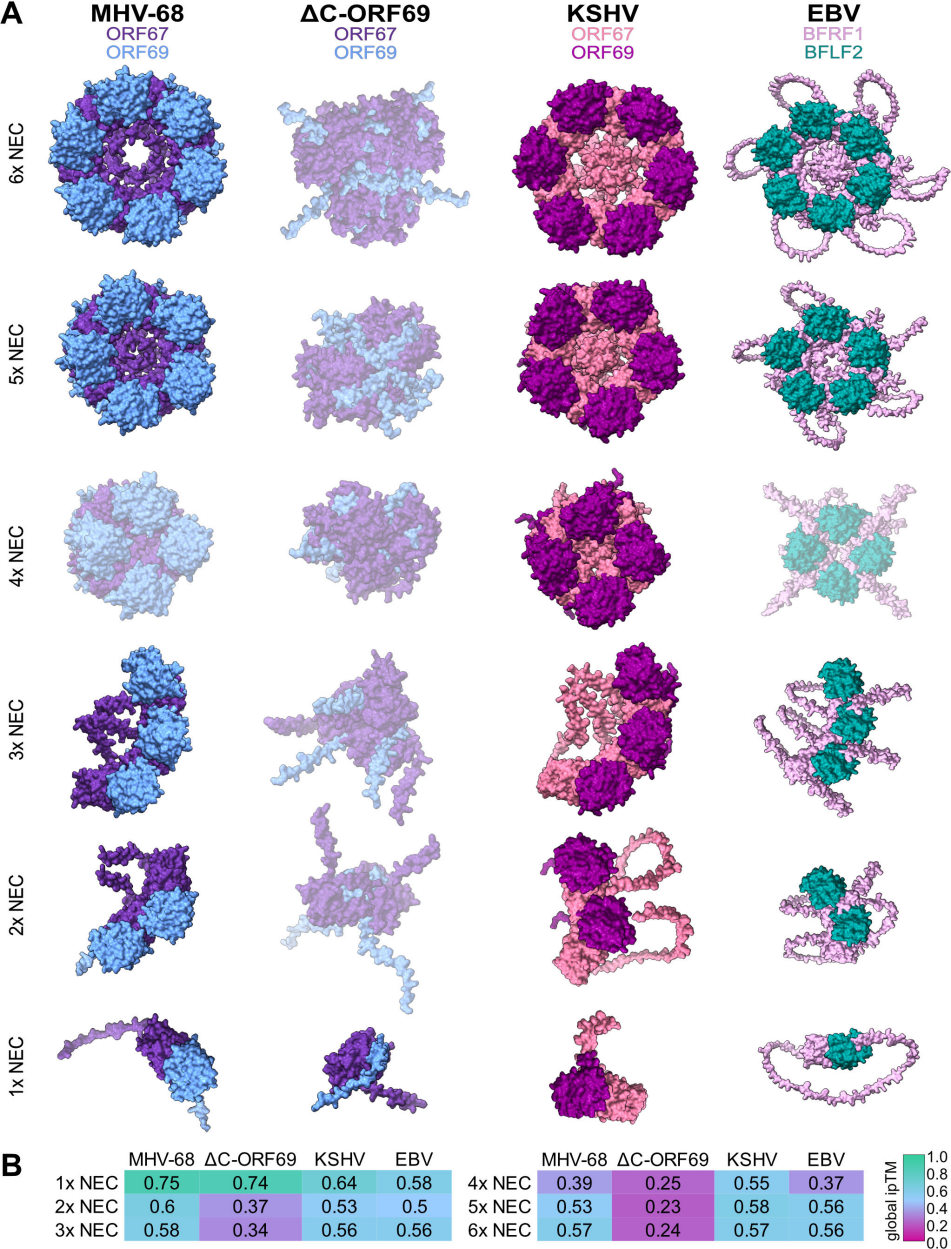
Predicted AlphaFold 3 structures of NEC oligomers of γ-herpesviruses. (**A**) *In silico* AlphaFold 3 structures for NEC-heterodimer oligomers (heterodimer to heterododecamer) in MHV-68 (ORF67 in purple, ORF69 in blue), ΔC-ORF69 (ORF67 in purple, ΔC-ORF69 in blue), KSHV (ORF67 in salmon, ORF69 in dark magenta), and EBV (BFRF1 in soft pink, BFLF2 in dark cyan). The following N-terminal residues are predicted to be disordered and omitted to aid visualization clarity: residues 1–47 of ORF69 MHV-68 ORF69 and ΔC-ORF69 ORF69, 1–38 of KSHV ORF69, and 1–78 of EBV BFLF2. Predictions with a global ipTM of <0.5 are grayed out. (**B**) Global ipTM scores for NEC-heterodimer oligomers (heterodimer to heterododecamer). The global ipTM score is color-coded from magenta (low confidence: 0) to green (high confidence: 1). The corresponding *in silico* structures for each virus and oligomer can be accessed at https://zenodo.org/records/15147695.

As there is no experimentally determined structure for the MHV-68 NEC (5), we utilized Alphafold 3 (32) to test *in silico* if our ΔC-ORF69 design is predicted to decouple heterodimer stability from higher-order assembly. First, we assessed the formation probability of the core heterodimers, MHV-68 ORF67-ORF69 and ORF67-ΔC-ORF69. Crucially, both models yielded high confidence scores with low Predicted Aligned Error (PAE) values (blue) combined with high pairwise interface predicted Template Modeling (ipTM) scores, indicating confident inter-chain interfaces (Fig. 2 and 3), suggesting that the retained N-terminal domain of ORF69 is predicted to be sufficient to maintain the critical hook-in-groove interaction with ORF67, *in silico* validating our design strategy.

In contrast, while both heterodimers were predicted to form stably, only the wild-type ORF67-ORF69 complex was predicted to support higher-order assembly required for lattice formation. The wild-type ORF67-ORF69 heterodimer was predicted to assemble into higher-order oligomers, up to a hexameric complex (Fig. 2 and 3). These assemblies were substantiated by favorable PAE heatmaps (blue) across the interfaces (Fig. 2A). Furthermore, the ipTM scores remained relatively high for trimeric, pentameric, and hexameric forms (Fig. 2B), consistent with the formation of higher-order complexes that form lattices (13).

In contrast, the ORF67-ΔC-ORF69 complex was predicted to remain heterodimeric. Attempts to model higher-order assemblies of the mutant resulted in PAE heatmaps dominated by high expected error (red) and low ipTM scores (Fig. 2). Importantly, AlphaFold will output a 3D structure even when confidence is low, which invalidates the structures depicted for the mutant in Fig. 3. This loss of interface confidence indicates that the mutant physically lacking the C-terminal domains likely does not form lattices, agreeing with previous structural studies on NEC oligomerization (11, 29, 30).

To further benchmark these predictions, we modeled the NEC multimers of the related γ-herpesviruses KSHV and EBV. KSHV consistently achieved good quality scores for both heterodimer and higher-order multimers (Fig. 2 and 3). Similarly, modeling of EBV-NEC oligomers identified relatively high interface confidence values, with the exception of heterooctamers, which in some cases exceeded the values of the heterodimer, consistent with previously reported conformational heterogeneity and structural plasticity in the crystal structures (29).

In summary, the structural predictions aligned with experimental data on related viruses and suggested that ΔC-ORF69 might act as a separation-of-function mutant by retaining the high-confidence interface necessary to interact with ORF67 but lacking the structural capability for higher-order oligomerization required for canonical nuclear egress.

### ΔC-ORF69 grows on non-complementing cells

Previous characterization of ORF69 relied on BAC-DNA transfection into human 293T cells, a method limited to analyzing early replication events (5). To assess the full replication dynamics across multiple rounds of infection, we generated viral stocks by reconstituting the ΔC-ORF69 BAC in NIH3T3 cells stably trans-complementing ORF69 (NIH3T3/ORF69+).

To determine whether ORF69 is strictly essential for spread, we infected non-complementing NIH3T3 cells. Contrary to the expectation of a null phenotype, ΔC-ORF69 produced visible plaques, indicating that the mutant retains the ability to spread, albeit with significantly reduced efficiency compared to the parental virus.

**Fig 4:**
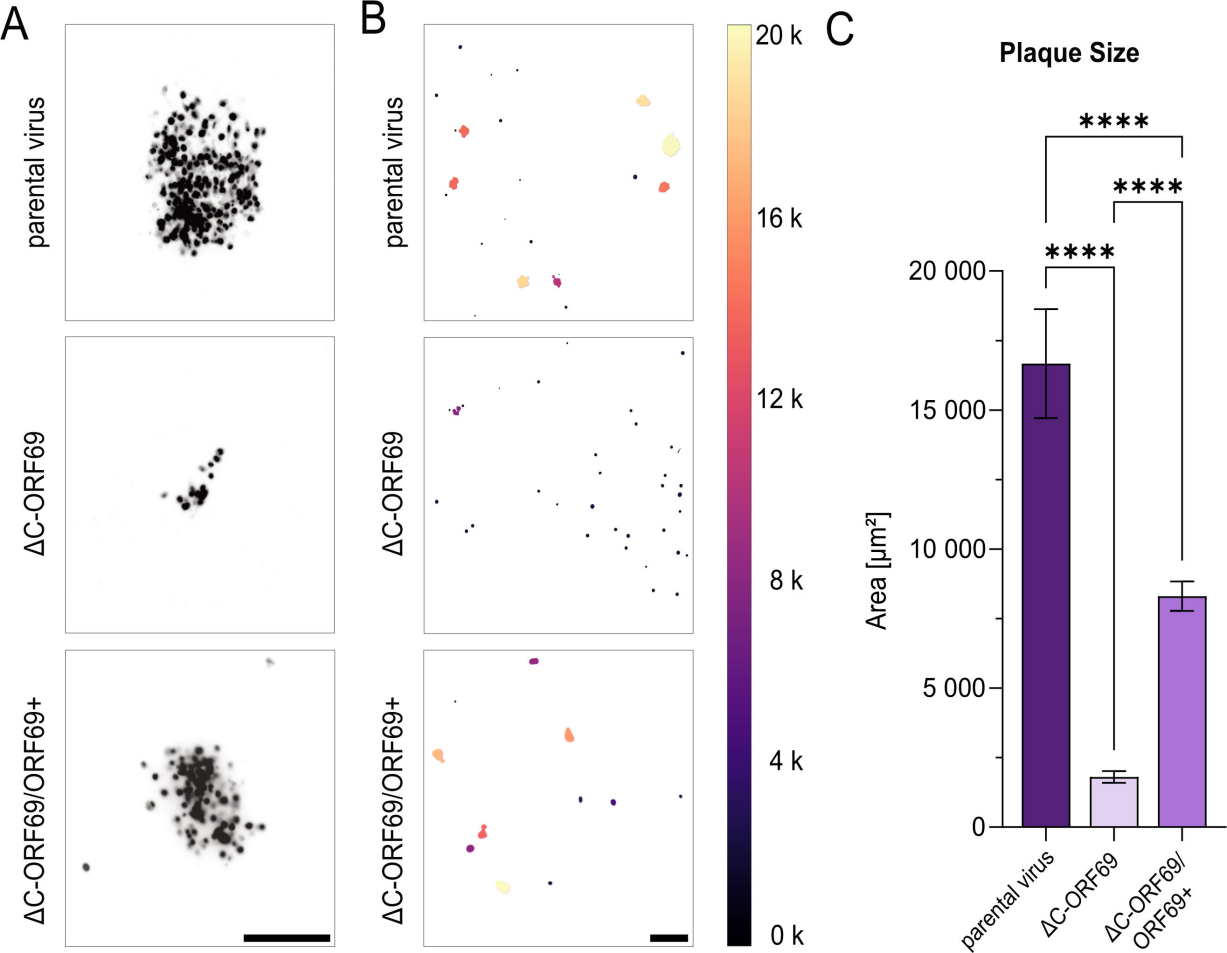
Comparison of the parental virus and ΔC-ORF69 plaque sizes. (**A**) Inverted fluorescent images of viral plaques at 4 dpi for the parental virus and ΔC-ORF69 on NIH3T3 cells, ΔC-ORF69 on NIH3T3 cells transcomplementing ORF69 (ΔC-ORF69/ORF69+). Scale bar: 200 µm. (**B**) Binary mask outputs from the image-processing pipeline used to measure plaque area (µm²), after preprocessing with a Gaussian blur to reduce noise and improve segmentation. Masks are color-coded according to plaque size, with the scale bar on the right. Excluded values are color-coded in black. Scale bar: 500 µm. (**C**) Mean area distribution of plaques represented as bar graphs of three independent biological replicates each for the parental virus (*n* = 516), ΔC-ORF69 (*n* = 182), and ΔC-ORF69/ORF69+ (*n* = 2,042), with 95% confidence intervals shown. ****, *P* < 0.0001 (Kruskal-Wallis test).

We quantified this defect using a custom image-processing pipeline to measure plaque area across 49 merged fields of view (FOV) (Fig. 4B). On non-complementing NIH3T3 cells, the mean plaque area of the parental virus was approximately ninefold larger than that of ΔC-ORF69. Infection of trans-complementing NIH3T3/ORF69+ cells significantly rescued the mutant phenotype, increasing the mean plaque area by approximately 4.5-fold compared to non-complementing cells (Fig. 4A and C). Still, plaques remained roughly two-fold smaller than parental levels, consistent with the notion that constitutive stable expression does not reproduce the native expression level and kinetics of viral proteins during infection.

These data demonstrate that ΔC-ORF69 is capable of residual spread in the absence of a functional NEC, which implies the existence of an alternative, albeit inefficient, way of facilitating capsid egress.

### ΔC-ORF69 does not acquire compensatory mutations during serial passaging

Given the critical role of NEC proteins in herpesvirus spread (1), the observation of plaque formation in ΔC-ORF69 infection of non-complementing cells necessitated an exclusion of parental virus contamination or genetic reversion. First, we analyzed the phenotypic characteristics of the mutant. Since the parental virus exhibits a ≈9-fold larger plaque area (Fig. 4), even minor contamination would result in rapid overgrowth and the appearance of large plaque outliers. However, our automated quantification of thousands of plaques across multiple replicates showed a tight distribution of small plaques with no such large outliers, arguing against the presence of a replication-competent parental virus.

**Fig 5:**
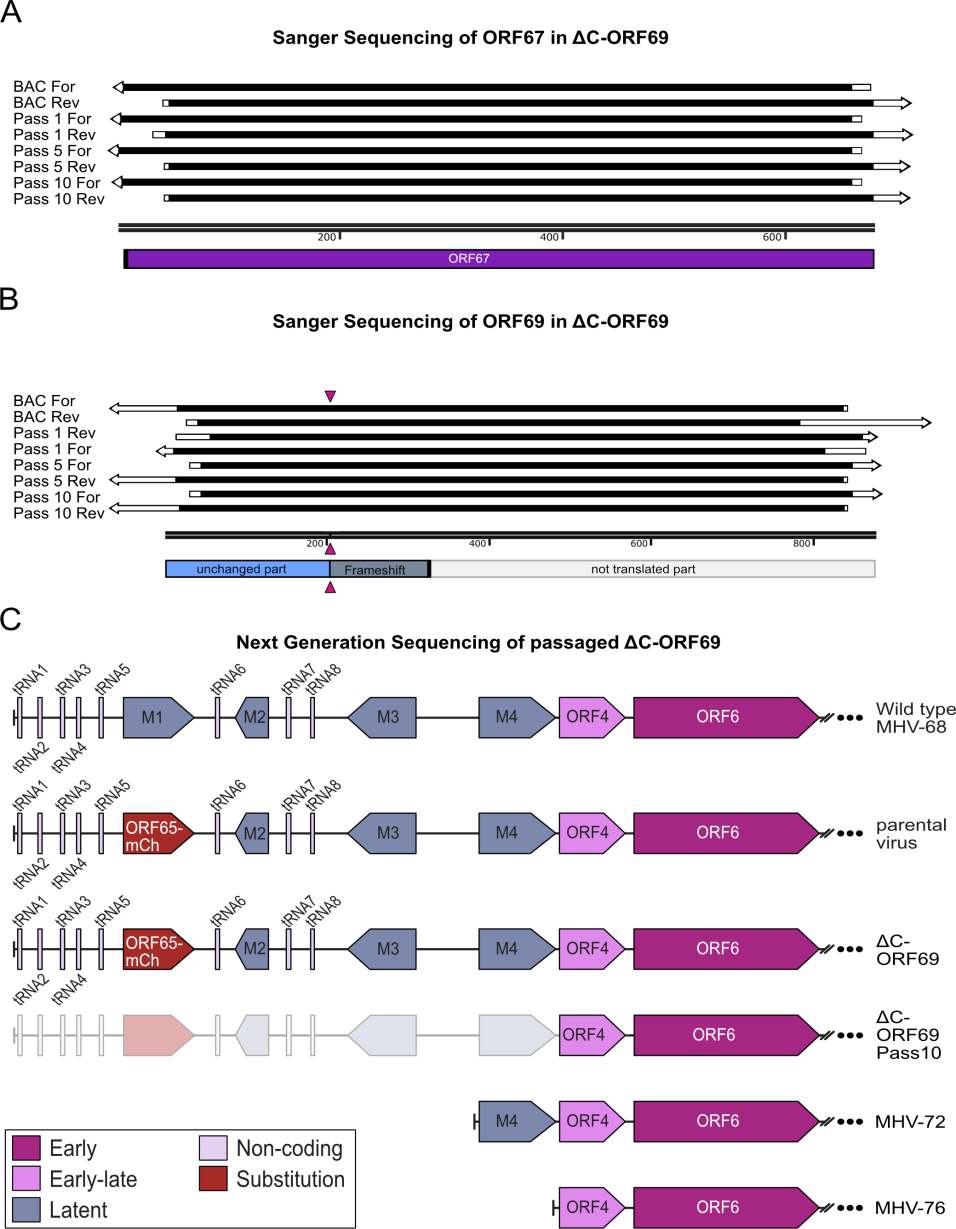
Validation of ORF67 and ORF69 via Sanger sequencing and NGS-based genome-wide comparison of the ΔC-ORF69 Pass 10. (**A and B**) Sequence alignments of ORF67 (**A**) and ORF69 (**B**) from the ΔC-ORF69 virus at different passages: BAC DNA (BAC), passage 1 (Pass 1), passage 5 (Pass 5), and passage 10 (Pass 10). Both forward and reverse strands were Sanger sequenced for each sample. (**A**) Sequence alignment of ORF67 in ΔC-ORF69 across all passages. ORF67 remained unaltered across all passages. Below the alignment, a schematic representation of the ORF67 coding sequence is shown in purple. (**B**) Sequence alignment of the mutated ORF69 in ΔC-ORF69 across all passages. A schematic representation of the ORF69 coding sequence is shown: the intact coding region in blue, the frameshift region in blue-gray, the non-translated downstream sequence in light gray, and the position of the mutation marked with pink triangles. Arrows indicate alignment quality: black represents complete identity with the reference; white indicates mismatches or low quality of the sequencing start and ends. (**C**) The panel compares the NGS-sequenced genome of ΔC-ORF69 Pass 10 (this study) with wild-type MHV-68, the parental virus (this study), ΔC-ORF69 (this study), and the field isolates MHV-72 and MHV-76. The ORF65-mCherry tag replacing the M1 gene is highlighted in red. The ΔC-ORF69 Pass 10 mutant exhibits a 9 kb deletion at the 5′ end of the genome, encompassing all eight tRNAs and the M1–M4 genes, shown as grayed out. This deletion is similar to those observed in field isolates MHV-72 and MHV-76. Non-coding tRNAs are depicted in lavender, latent genes in steel blue, early-late genes in orchid, and early genes in deep magenta.

Second, we validated the genotype. Sanger sequencing of the ORF69 and ORF67 genomic regions in the viral stocks confirmed that the specific frameshift mutation was maintained (Fig. 5A and B). Crucially, the chromatograms showed clean traces without the double peaks expected in a mixed population, confirming that the phenotype is driven by the intended mutation rather than a revertant or contaminant. Having established that the residual spread is intrinsic to the ΔC-ORF69 mutant, we next investigated whether the virus utilizes adaptive mechanisms to enable the limited spread. In the α-herpesvirus Pseudorabies virus (PrV), NEC-deficient mutants (ΔUL31, ΔUL34) accumulate specific compensatory mutations upon extensive serial passaging that facilitate NEBD, thereby restoring viral titers (18, 19). To determine whether ΔC-ORF69 can follow a similar adaptive trajectory, we passaged the virus 10 times (Pass 10) on non-complementing NIH3T3 cells. While the cytopathic effect (CPE) accelerated by passage 10, the virus unexpectedly lost mCherry fluorescence. We re-evaluated ORF67 and ORF69 via Sanger sequencing in the Pass 5 and Pass 10 populations, confirming that the NEC loci remained unaltered and free of compensatory mutations (Fig. 5A and B). To identify the genomic basis for the accelerated CPE and loss of fluorescence, we performed whole-genome sequencing for Pass 10 (Illumina MiSeq). Analysis revealed a single major genomic alteration: a 9 kb deletion at the 5′ end of the genome encompassing the M1–M4 genes and all eight tRNAs. PCR and Sanger sequencing of the deletion boundary confirmed that the downstream ORF4 remained intact (Fig. 5C, ΔC-ORF69 Pass 10). This deletion accounts for the loss of fluorescence, as the parental virus harbors a duplicated mCherry-tagged ORF65 within the dispensable M1 locus (25). The loss of this exogenous sequence could be one explanation for the accelerated CPE. Furthermore, spontaneous deletions of the 5′ end are a documented adaptation of MHV-68 to tissue culture (33) and are characteristic of field isolates such as MHV-72 (34) and MHV-76 (35, 36). Crucially, unlike PrV mutants (19), ΔC-ORF69 did not acquire specific compensatory mutations in glycoproteins or nuclear rim factors to facilitate alternative egress. The observed accelerated CPE is therefore attributable either to general tissue-culture adaptation (the 5′ deletion) or increased viral fitness through loss of exogenous sequence, such as the ORF65-mCherry fusion, like observed for plant viruses (37), rather than a specific restoration of the nuclear egress pathway.

### ΔC-ORF69 exhibits normal capsid assembly and DNA packaging

Having excluded genetic reversion to the wild-type ORF69 sequence, we sought to define the stage at which ΔC-ORF69 egress seems to be impaired but not abolished. Besides the role of translocating capsids from the nucleus to the cytoplasm, additional roles in capsid assembly and DNA packaging have been proposed for ORF69 homologs in other herpesviruses. HSV-1 UL31 is required for efficient DNA cleavage (38), MCMV M53 plays an accessory role in packaging (39), and deletion of EBV BFLF2 reduces the production of genome-filled C-capsids and increases levels of A- and B-capsids in the nucleus while lytic DNA replication is not altered (40).

**Fig 6:**
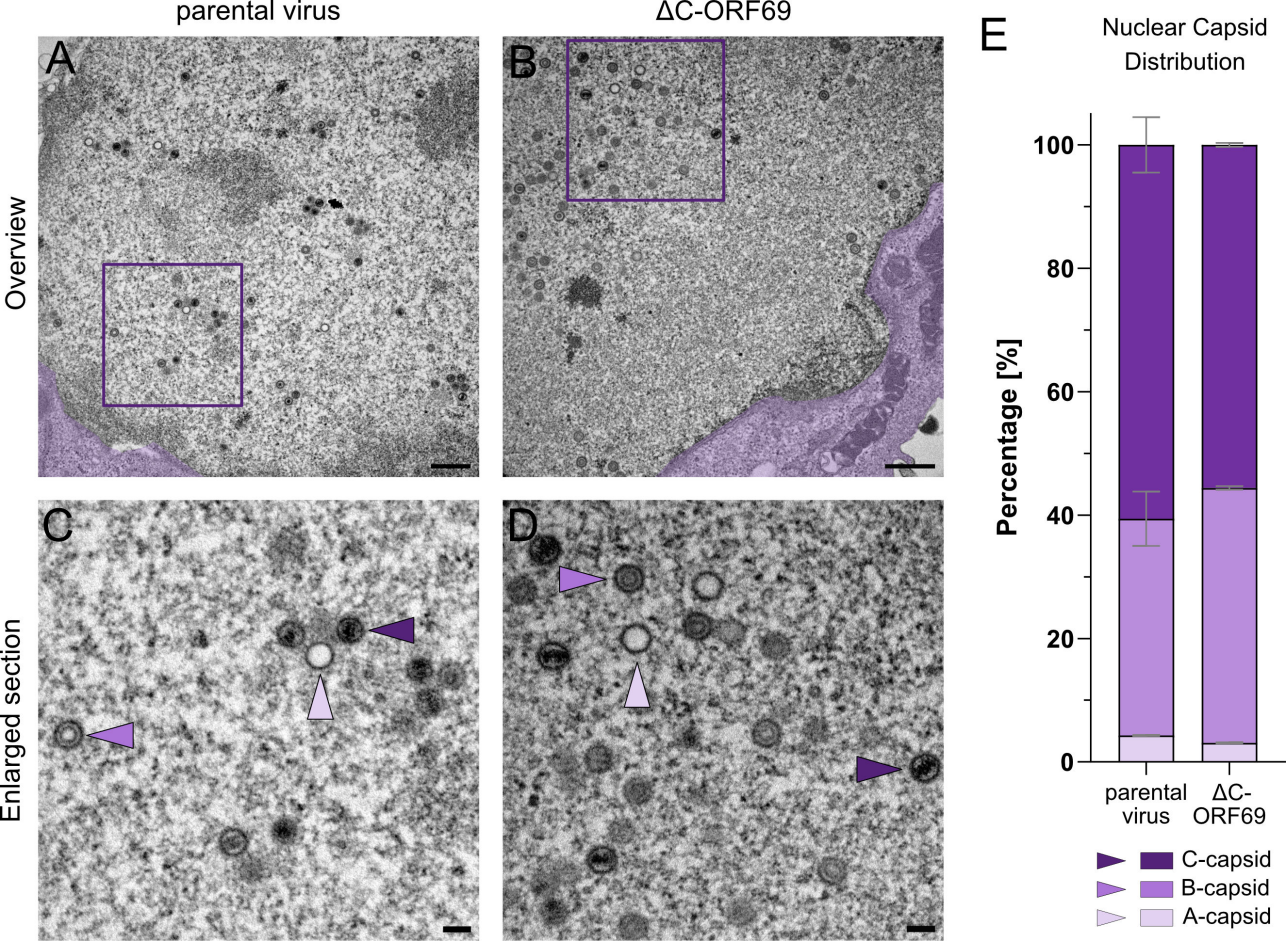
Quantification of nuclear capsid forms in the parental virus and ΔC-ORF69. (**A and B**) Representative TEM images of the nucleus in the parental virus-infected at 1 dpi and ΔC-ORF69-infected NIH3T3 cells at 4 dpi. The adjacent cytoplasm is false-colored in purple. Scale bar: 500 nm. (**C and D**) Enlarged views of the nucleoplasm from the boxed regions in panels A and B, highlighting the three distinct capsid types: C-capsids (DNA-filled, indigo arrowheads), B-capsids (scaffold-containing, orchid arrowheads), and A-capsids (empty, lavender arrowheads). Scale bar: 100 nm. (**E**) Quantification of the relative proportion (%) of nuclear capsid types. Bars represent mean ± SD from two independent replicates. Parental virus: n_cells_ = 14, n_FOV_ = 455, n_capsids_ = 5,527, ΔC-ORF69: n_cells_ = 10, n_FOV_ = 330, n_capsids_ = 4,761.

To determine whether the ΔC-ORF69 mutation exhibits these capsid maturation phenotypes and to localize the site at which capsid egress is impaired, we performed quantitative transmission electron microscopy (TEM), quantifying over 10,000 capsids on serially sectioned nuclei, as previously done for EBV (40). Morphologically, parental and ΔC-ORF69-infected nuclei were indistinguishable and showed classical hallmarks of herpesvirus-infected nuclei. Both displayed regions of darker, condensed chromatin, similar nuclei shape, and no clear disruption of the nuclear envelope (Fig. 6A and B). Capsid assembly appeared robust in the mutant, with A-, B-, and C-capsids evenly distributed in loose clusters throughout the nucleoplasm (Fig. 6C and D).

Quantitative classification of more than 10,000 capsids into A-, B-, and C-capsids revealed comparable capsid type distributions between the parental virus and the ΔC-ORF69 mutant, indicating that capsid assembly and DNA packaging are not detectably altered via TEM under these conditions (Fig. 6E). In both parental and mutant infections, genome-filled C-capsids constituted the majority population (Parental: 60.6% ± 4.5%; ΔC-ORF69: 55.9% ± 0.3%). While the mutant exhibited a slight increase in scaffold-containing B-capsids (41.3% ± 0.3% vs 35.1% ± 4.4%), the proportion of empty A-capsids remained negligible (<5%) in both conditions.

These data show that, unlike its EBV homolog (40), assembly of capsids and DNA packaging are not severely altered in the ΔC-ORF69 mutant. Since the nuclei contained an abundance of mature, genome-containing C-capsids, the observed reduction in viral spread and progeny production is unlikely to result from impaired capsid assembly but instead points to a defect at a later stage of the viral life cycle. Consequently, we next examined cytoplasmic capsids to assess whether nuclear egress was impaired.

### ΔC-ORF69 blocks capsid export, while the parental virus selectively exports C-capsids

To determine whether capsids could bypass the NEC via non-canonical routes, such as NEBD as shown for PrV and HSV-1 (18–20, 41) or dilated nuclear pores, as implied for HSV-1 (21), in the absence of a functional C-terminus, we examined the cytoplasmic composition of infected cells using TEM.

**Fig 7:**
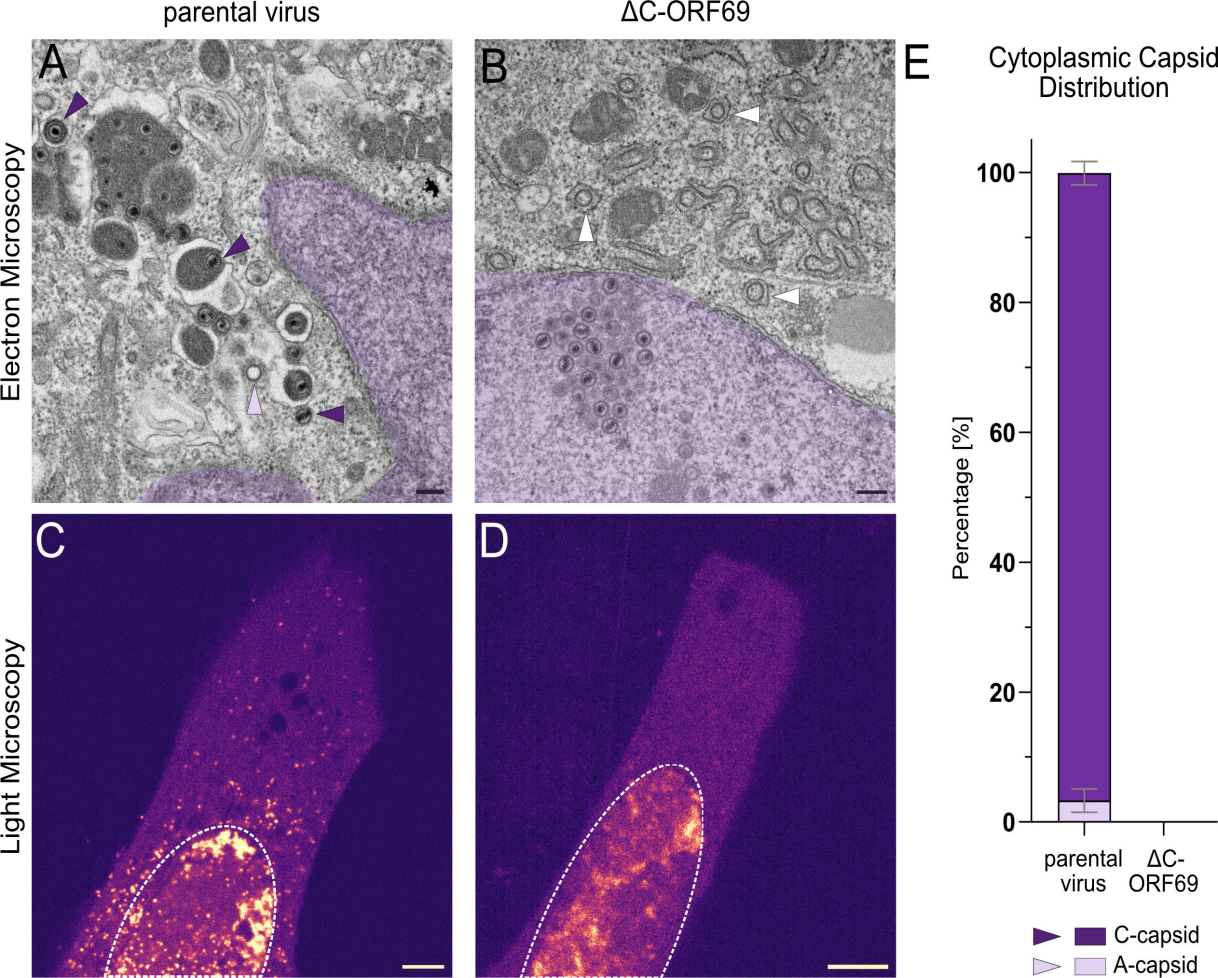
Analysis of cytoplasmic capsids in the parental virus and in ΔC-ORF69. (**A and B**) Representative TEM images of the cytoplasm in the parental virus-infected at 1 dpi and ΔC-ORF69-infected NIH3T3 cells at 4 dpi. The adjacent nucleus is false-colored in purple. (**A**) C-capsids (DNA-filled, indigo arrowheads) and A-capsids (empty, lavender arrowheads) in the cytoplasm. (**B**) Vesicular structures (white arrowheads). Scale bar: 200 nm. (**C and D**) Representative overview live-cell images of the NIH3T3 cells infected with the parental virus (**C**) or ΔC-ORF69 (**D**) at 1 dpi. The nucleus is marked with a dotted line. The images were color-coded with the “mpl-magma” LUT in Fiji ImageJ, where low-intensity signals are shown in indigo and high-intensity signals in yellow-white. Scale bar: 5 µm. (**E**) Quantification of the relative proportion (%) of different cytoplasmic capsid types in TEM. Bars represent mean ± SD from two independent replicates of the parental virus. Parental virus: n_cells_ = 14, FOV: 219, n_capsids_ = 1956. No capsids were present in the cytoplasm of ΔC-ORF69 at 4 dpi across 10 examined cells.

In parental virus-infected cells, the cytoplasmic morphology indicated active, selective egress. The cytoplasm predominantly contained C-capsids (Fig. 7A, indigo arrowheads), with only a minor fraction of empty A-capsids (Fig. 7A, lavender arrowhead) and a complete absence of B-capsids in all FOV examined. We also observed distinct, electron-dense proteinaceous structures, likely representing tegument reservoirs involved in maturation, likely secondary envelopment. Indeed, we captured C-capsids at various stages of this process: a naked C-capsid (Fig. 7A, bottom indigo arrowhead), a C-capsid budding into the tegument-rich structure (middle), and a fully tegumented and enveloped capsid (top). Quantitative analysis across multiple FOVs confirmed this strong selection bias: 96.6% ± 1.8% of cytoplasmic capsids were DNA-filled C-capsids (Fig. 7E).

In contrast, no cytoplasmic capsids were detected in ΔC-ORF69-infected cells across all examined fields of view (n_cells_ = 10; n_FOV_ = >200). Live-cell imaging at 1 dpi substantiated this lack of cytoplasmic capsids. While the parental virus displayed numerous fluorescent puncta in the cytoplasm corresponding to egressed capsids (Fig. 7C), the ΔC-ORF69 signal was strictly confined to the nucleus (Fig. 7D).

Notably, in ΔC-ORF69-infected cells, vesicular structures were identified by TEM at the nuclear periphery within ER-associated membrane compartments, precluding a clear distinction between perinuclear and cytoplasmic localization. The vesicular structures had a diameter of approximately 100 nm and lacked an associated viral capsid (Fig. 7B, white arrowhead). This phenotype was not observed in cells infected with the parental virus. Similar vesicular structures have been reported for KSHV ORF67/ORF69 overexpression systems in Sf21 cells (42). Luitweiler et al. (43) quantified the subcellular localization of KSHV ORF67/ORF69-induced membrane phenotypes in Sf21 cells and found that vesicular structures formed in the presence of both ORF67 and ORF69 were predominantly intranuclear, with only rare perinuclear or cytoplasmic occurrences. Similar vesicular structures were also observed for PrV, although they were restricted to the nuclear and perinuclear spaces (44). In this context, the consistent accumulation of comparable vesicular structures in ΔC-ORF69-infected cells represents a clear shift in localization relative to the mostly nuclear ORF67/ORF69 coexpression data. Thus, while the vesicular morphology aligns with previously described NEC-associated membrane remodeling, their localization in ΔC-ORF69 infection suggests that the spatial organization of this remodeling differs under infection conditions when ORF69 is C-terminally truncated (43).

### Absence of extracellular particles is consistent with a defect in nuclear egress in ΔC-ORF69

To verify that the predominantly C-capsid from the cytoplasmic fraction in the parental virus represents the capsids egressing from the cell and to verify that the absence of cytoplasmic capsids in the mutant reflected an impairment in egress rather than rapid release, we quantified viral particles in the extracellular space of embedded cells.

**Fig 8:**
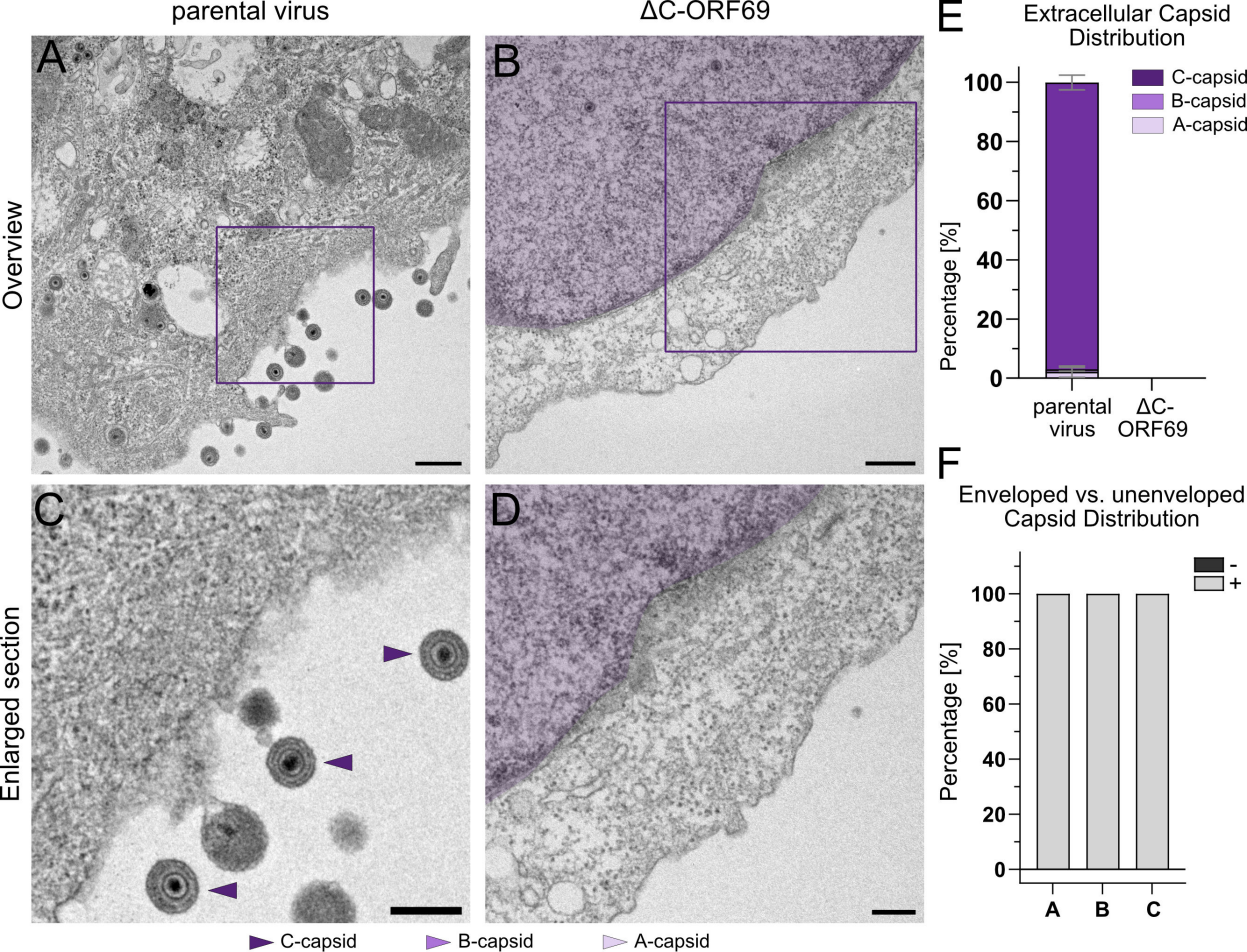
Analysis of extracellular capsids in the parental virus and in ΔC-ORF69. (**A and B**) Representative TEM images of the extracellular space in the parental virus-infected at 1 dpi and ΔC-ORF69-infected NIH3T3 cells at 4 dpi. Scale bar: 500 nm. (**C and D**) Enlarged views of the extracellular space with adjacent cytoplasm from the boxed regions (**A and B**). The adjacent nucleus (**B and D**) is false-colored in purple. (**E**) Quantification of the relative proportion (%) of different extracellular capsid types. Bars represent mean ± SD from two independent replicates of the parental virus. Parental virus: FOV: 68, n_capsids_ = 424. No extracellular capsids were present in the cytoplasm of ΔC-ORF69 at 4 dpi. (**F**) Quantification of the relative proportion (%) of enveloped (+) versus unenveloped (−) capsid types in the extracellular space of the parental virus using the same data set as analyzed in panel E. Bars represent mean ± SD from two independent replicates.

Consistent with the cytoplasmic data, no extracellular capsids were detected in ΔC-ORF69-infected cells (Fig. 8B and D). Conversely, the extracellular space of parental virus-infected cells contained abundant viral particles (Fig. 8A and C). Quantification mirrored the cytoplasmic selectivity: 96.9% ± 2.5% were C-capsids, with only negligible fractions of A- (2.2% ± 1.9%) or B-capsids (0.8% ± 0.6%) (Fig. 8E). Notably, all of these extracellular particles were enveloped (Fig. 8F). The presence of rare extracellular A- and B-capsids, which were also enveloped, suggests that while the NEC imposes a strict checkpoint at the nuclear envelope, the secondary envelopment machinery in the cytoplasm may be less selective.

### Late-stage NEBD enables non-selective capsid egress

**Fig 9:**
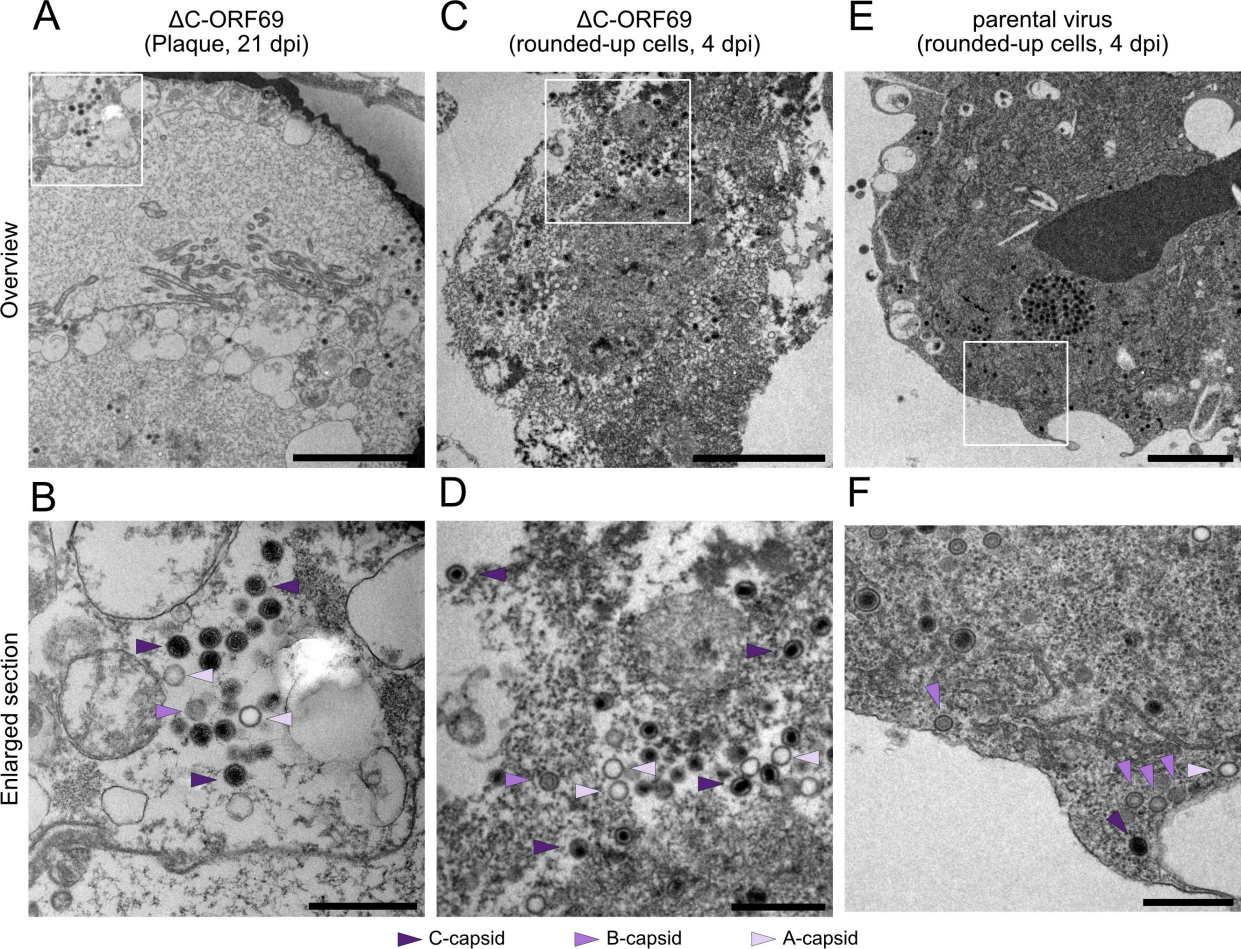
Analysis of late-stage cytoplasmic capsids in ΔC-ORF69 and the parental virus. (**A, C, E**) Representative TEM overview images of late-stage infected NIH3T3 cells. (**B, D, F**) Enlarged views of the boxed regions in (**A, C, E**), respectively, highlight cytoplasmic capsids. A-, B-, and C-capsids are indicated by lavender, orchid, and indigo arrowheads, respectively. Scale bars: 2 µm (**A, C, E**); 500 nm (**B, D, F**). (**A and B**) ΔC-ORF69-infected cells at 21 dpi within a plaque with a compromised nucleus and cytoplasmic capsids. (**C and D**) ΔC-ORF69-infected cells at 4 dpi embedded in a cellulose tube, showing cytoplasmic capsids. (**E and F**) Parental-virus-infected cells at 4 dpi embedded in a cellulose tube, showing different capsid forms in the cytoplasm.

Since ΔC-ORF69 eventually forms small plaques (Fig. 4), we reasoned that capsids must escape the nucleus through an alternative mechanism. To capture these events, we analyzed ΔC-ORF69-infected cells at very late stages of infection. We specifically processed plaques at 21 dpi because they consist of multiple infection stages, with cells surrounding the lysed center of the plaque infected for longer than those at the periphery, thereby increasing the likelihood of detecting rare late-stage phenotypes. However, these 21 dpi time points were only used for illustrative purposes and were not included in quantitative assessments. Moreover, it is not possible to determine the precise infection duration of individual cells within these plaques. Indeed, we were able to observe rounded-up cells with compromised nuclear integrity (Fig. 9A). Unlike the selective export seen in the parental virus, these cells contained cytoplasmic capsid clusters with a non-selective mix of A-, B-, and C-capsids (Fig. 9B).

As small plaques in mutant virus samples were already visible at 4 dpi (Fig. 4A), we reasoned that a subpopulation of cells may reach this compromised state earlier in infection. Consistent with this interpretation, we detected NEBD not only in 21 dpi plaques but also at 4 dpi in ΔC-ORF69-infected cells. However, these late-stage cells often detach and are typically lost during standard preparation. To capture this specific population, we collected floating cells at 4 dpi from both the supernatant and after gentle rinsing, concentrated them, and encapsulated them in cellulose capillary tubes to enable embedding of non-adherent cells without loss during subsequent staining and washing steps (45). This approach allowed us to identify individual ΔC-ORF69-infected cells with compromised nuclei (Fig. 9C) that, similar to the 21 dpi samples, contained all three capsid forms in the cytoplasm (Fig. 9D). Importantly, we observed similar NEBD and non-selective leakage of capsids in late-stage infected cells infected with the parental virus (Fig. 9E and F), arguing for a general phenotype late in infection.

Collectively, these observations indicate that in the absence of a functional NEC, capsids are retained in the nucleus until late-stage infection, where they leak into the cytoplasm only upon loss of nuclear integrity. While NEBD-associated capsid release is observable in cells infected for 4 days and in cells part of plaques that expanded for 21 days, we cannot conclude that the mode of nuclear escape observed is identical, even though the individual cell infection cycles still likely span only a few days. Still, these data are consistent with the idea that capsid release at late stages may occur through passive leakage associated with nuclear rupture, as observed for Adenoviruses (46).

### ΔC-ORF69 releases rare enveloped C-capsids, explaining residual infectivity

**Fig 10:**
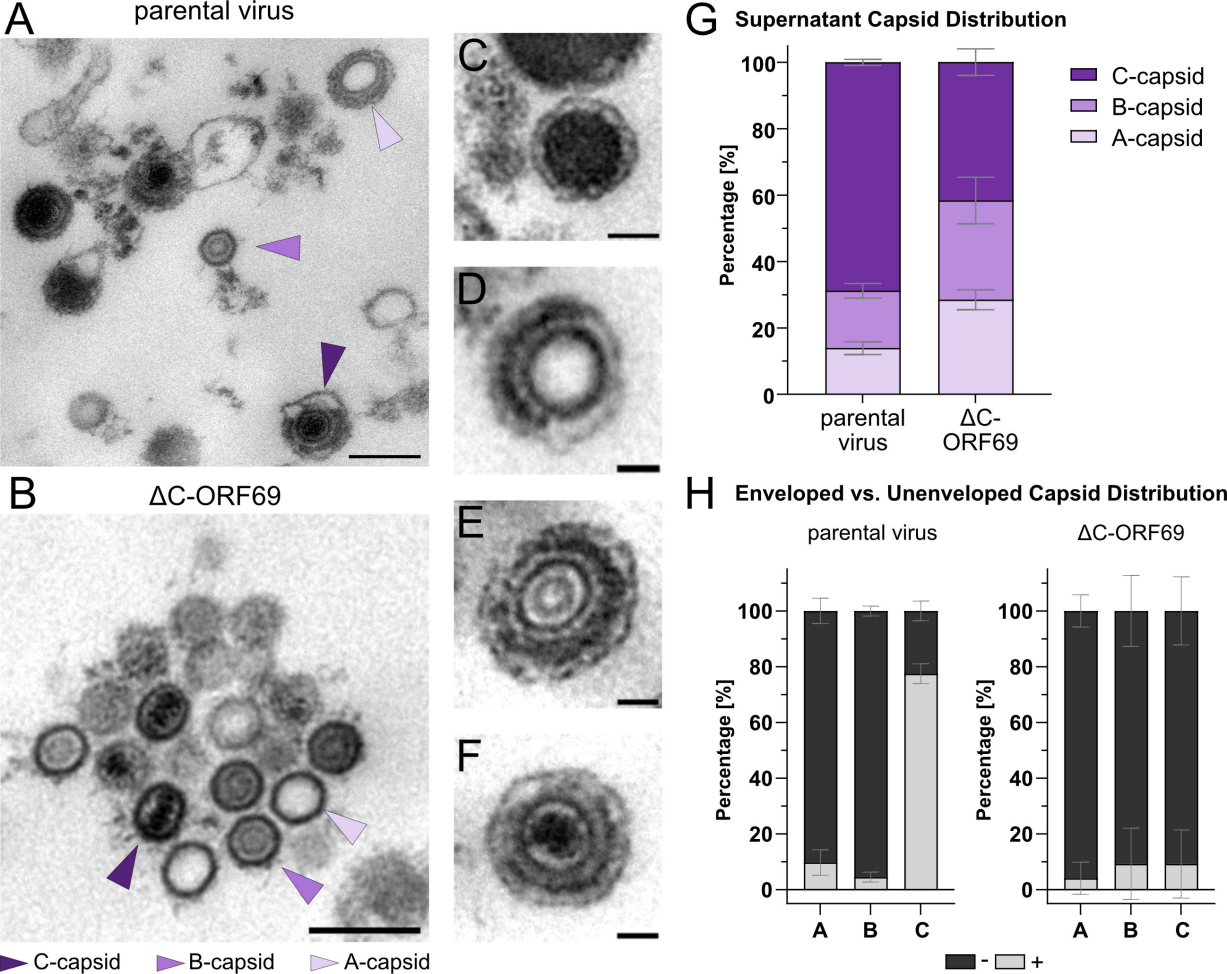
Capsid forms in the concentrated supernatant of the parental virus and ΔC-ORF69. (**A–F**) Overview TEM images of particles from pelleted supernatant particles. (**A**) Enveloped and unenveloped capsids from the parental virus displaying all three capsid forms: DNA-filled C-capsids (indigo arrowhead), scaffold-containing B-capsids (orchid arrowhead), and empty A-capsids (lavender arrowhead). (**B**) A cluster of naked capsids in ΔC-ORF69. (**C–F**) Examples of enveloped particles from ΔC-ORF69: (**C**) dense body, (**D**) enveloped A-capsid with an incomplete tegument layer, (**E**) enveloped B-capsid, and (**F**) enveloped C-capsid. (**G**) Quantification of the relative proportion (%) of A-, B-, and C-capsids in the supernatant of the parental virus (4 dpi) or ΔC-ORF69 (3 dpi). Bars represent mean ± SD from two independent biological replicates. Parental virus: n_Grids_ = 6, n_FOV_ = 64, n_capsids_ = 1,598. ΔC-ORF69: n_Grids_ = 6, n_FOV_ = 178, n_capsids_ = 1,550. (**H**) Quantification of the proportion (%) of enveloped (+) versus unenveloped (−) capsids, using the same data set as analyzed in panel G.

To illuminate the ultrastructural basis of the residual infectivity observed in plaque assays, we analyzed extracellular viral particles to determine if capsids released via NEBD could acquire an envelope. We concentrated viral material from the supernatant of ΔC-ORF69-infected cells (3 dpi) and parental virus-infected cells (4 dpi) and encapsulated the pellets in cellulose capillary tubes to preserve structural integrity where needed (45). Electron microscopy of the parental virus supernatant revealed a heterogeneous population containing enveloped and unenveloped forms of all three capsid types (Fig. 10A). In contrast, the ΔC-ORF69 supernatant was characterized by large clusters of naked capsids (Fig. 10B) and electron-dense vesicles lacking capsids, likely representing dense bodies (Fig. 10C), which are non-infectious particles primarily of tegument and lacking a capsid. However, we identified enveloped A-, B-, and, crucially, C-capsids within the samples (Fig. 10D through F). Although rare, the presence of these mature, enveloped particles confirms that capsids released via NEBD can occasionally undergo secondary envelopment, providing a mechanistic explanation for the mutant’s ability to spread.

Quantitative analysis underscored both the loss of selectivity and the inefficiency of this process. While the parental virus supernatant late in infection was dominated by C-capsids (68.8% ± 0.9%), albeit with a reduced proportion compared to the observation in the cytoplasm and the extracellular space at 1 dpi, the ΔC-ORF69 supernatant exhibited a non-selective distribution. C-capsids constituted the largest fraction (41.7% ± 4.0%), but B-capsids (29.9% ± 7.0%) and A-capsids (28.5% ± 3.0%) were present at similarly high levels (Fig. 10G), consistent with the indiscriminate leakage of nuclear contents. Moreover, assessment of envelopment efficiency (Fig. 10H) revealed a stark contrast. In parental virus samples, envelopment was highly efficient for C-capsids (77.5% ± 2.5% enveloped). In the ΔC-ORF69 mutant, however, envelopment was highly inefficient across all capsid types, with only a small fraction of C-capsids (9.3% ± 8.7%) acquiring envelopes. This low frequency, combined with morphological irregularities such as partial tegumentation (Fig. 10D), indicates that while secondary envelopment is possible during late-stage NEBD, it is likely functionally impaired.

Taken together, these data suggest that in the absence of a functional NEC, capsids that escape from compromised nuclei can undergo secondary envelopment, albeit with very low efficiency. Importantly, the detection of enveloped A- and B-capsids in both parental and mutant supernatants indicates that the cytoplasmic secondary envelopment machinery lacks strict capsid-type specificity and can envelope any capsid form that gains access to the cytoplasm, thereby highlighting the NEC as the key determinant of C-capsid selectivity during nuclear egress. This supports a model in which the NEC serves as the primary and most critical capsid control checkpoint for selecting genome-filled C-capsids.

### Inhibition of CDK-dependent pathways reduces the residual spread of ΔC-ORF69

Previous studies had shown that NEC-deficient PrV mutants (PrV ΔUL31, PrV ΔUL34) regain replication competence by exploiting CDK-dependent NEBD to bypass the nuclear barrier (19, 47). To determine if a similar host-dependent mechanism facilitates the residual spread of ΔC-ORF69, we treated infected cells with small-molecule inhibitors targeting key cell cycle and signaling regulators.

We selected Roscovitine to target CDKs, as it potently inhibits cdc2/cyclin B (IC50 = 0.65 µM), cdk2/cyclin A (0.7 µM), cdk2/cyclin E (0.7 µM), and cdk5/p53 (0.16 µM) while showing considerably weaker activity against ERK1/2 (IC50 = 34 µM and 14 µM) (48). Roscovitine has previously been shown to reduce wild-type MHV-68 replication (49). For comparison, we used U0126, a highly selective inhibitor of the MAPK/ERK pathway (IC50 ≈0.06–0.07 μM for MEK1/2) (50), which has only a minor effect on MHV-68 replication (51).

**Fig 11:**
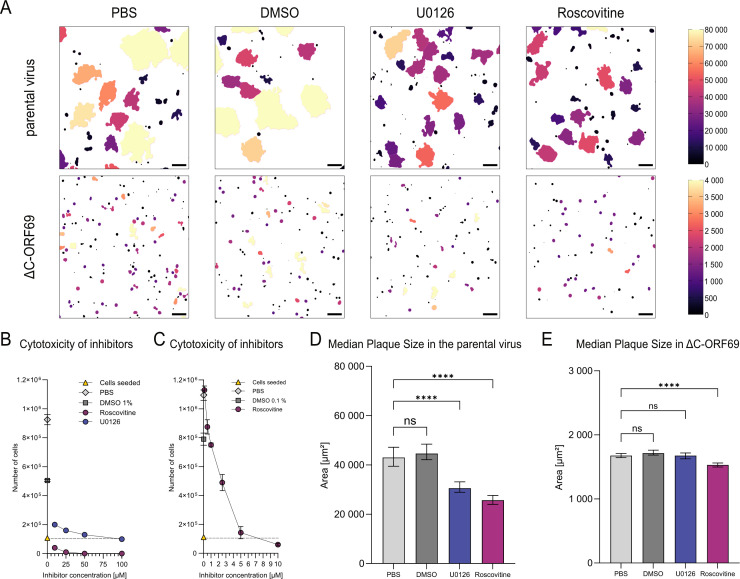
Effect of inhibitors on plaque size in the parental virus and ΔC-ORF69. (**A**) Binary mask outputs from the image-processing pipeline, following Gaussian blur preprocessing to reduce noise and enhance segmentation, showing plaque area (µm²) for the parental virus (upper panel) and ΔC-ORF69 (lower panel) under PBS, DMSO, U0126, and Roscovitine treatment. The upper panel is color-coded on a scale (0–80,000 µm²), and the ΔC-ORF69 panel uses a 0–4,000 µm² scale. Scale bar: 200 µm. (**B and C**) NIH3T3 cells treated with increasing concentrations of U0126 and Roscovitine. Cell numbers were assessed at 3 dpi. Data with error bars represent the mean ± SD of two technical replicates. Single measurements are shown without error bars. (**B**) High concentrations of inhibitors (10–100 µM). (**C**) Low concentrations of Roscovitine (0.1–10 µM). The gray dotted line represents the initial seeding density. Colors: Cells seeded (yellow), PBS (light gray), DMSO controls (dark gray, concentration: B: 1%, C: 0.1%), Roscovitine (dark magenta), and U0126 (dark slate blue). (**D**) Quantification of parental virus plaque area (µm²) in four independent experiments treated with 50 µM U0126 or 5 µM Roscovitine, alongside PBS and 0.5% DMSO (in PBS) controls (PBS_parental_ *n* = 1,130, DMSO_parental_ *n* = 1,269, U0126_parental_ *n* = 1,318, Roscovitine_parental_ *n* = 1,247). A size threshold of >3,000 µm² was applied, ****, *P* < 0.0001; ns, not significant *P* > 0.05 (Kolmogorov-Smirnov test). Bars represent the median with 95% confidence intervals. (**E**) Quantification of ΔC-ORF69 plaque area (µm²) in four independent experiments treated with 50 µM U0126 or 5 µM Roscovitine, alongside PBS and 0.5% DMSO (in PBS) controls (PBS_ΔC-ORF69_ *n* = 3,817, DMSO_ΔC-ORF69_ *n* = 3,274, U0126_ΔC-ORF69_ *n* = 2,157, Roscovitine_ΔC-ORF69_ *n* = 2,041). A size threshold of >1,000 µm² was applied, ****, *P* < 0.0001; ns, not significant *P* > 0.05 (Kolmogorov-Smirnov test). Bars represent the median with 95% confidence intervals.

Prior to infection assays, we established optimal inhibitor concentrations (5 μM Roscovitine, 50 μM U0126). These concentrations effectively modulated cell cycle checkpoints, inducing mitotic arrest (Roscovitine) or G2 delay (U0126), while maintaining stable cell numbers and preserving overall viability throughout the testing period (Fig. 11B and C). Using our automated image-processing pipeline to quantify fluorescent plaques (Fig. 11A), we observed distinct sensitivities between the parental and mutant viruses. In parental virus infections, both inhibitors significantly reduced plaque size (Fig. 11D). The reduction observed with U0126 confirms that MEK1/2 signaling contributes to efficient wild-type spread, likely by optimizing the cellular environment for replication. In contrast, the ΔC-ORF69 mutant was insensitive to MEK1/2 inhibition, as the plaque size was unaffected by U0126 (Fig. 11E). This lack of additional effect suggests a functional relationship between ORF69 and MEK1/2 signaling. Alternatively, the severely reduced plaque size of the ΔC-ORF69 mutant may limit the extent to which further modulation of MEK1/2 signaling can measurably impact viral spread. Although the effect of Roscovitine was more pronounced in the parental virus compared to the respective control, its significant reduction of the already very small ΔC-ORF69 plaques suggests that CDK activity is critical for this residual viral propagation. While CDKs are known to be essential for viral replication, the continued sensitivity of the mutant suggests that this alternative egress route via NEBD may also rely on host factors regulated by cell-cycle kinases, such as those controlling nuclear envelope integrity. This aligns with the hypothesis that, in the absence of the NEC, the virus exploits host cell cycle machinery to escape from the nucleus.

## DISCUSSION

Our study demonstrates that the nuclear egress complex (NEC) of MHV-68 plays a crucial role in mediating C-capsid specificity during infection. The ΔC-ORF69 mutant retained a limited ability to spread in NIH3T3 cells, characterized by reduced efficiency and significantly smaller plaque sizes compared to the parental virus. Quantitative electron microscopy revealed that this C-terminal truncation of ORF69 abolished selective nuclear egress, resulting in the indiscriminate occurrence of A-, B-, and C-capsids in the cytoplasm late in infection. This contrasts sharply with parental virus infection, where genome-filled C-capsids are the predominant form exported. This suggests that the γ-herpesvirus NEC functions as a critical selective gate, facilitating the translocation of mature C-capsids while retaining immature or defective particles within the nucleus. This selective gate function must be considered in the context of the NEC’s well-established roles in membrane remodeling and capsid translocation.

The function of the NEC as a membrane remodeler (42–44) and a capsid translocator via an envelopment-de-envelopment mechanism is well established (52). In α-herpesviruses, the deletion of NEC components typically results in the intranuclear accumulation of capsids (16, 17, 38). Similar phenotypes have been reported for the γ-herpesvirus EBV, where the knockout of either BFLF2 or BFRF1 leads to strict nuclear retention (40). However, emerging evidence suggests that the NEC is not absolutely required for detectable infectivity, as residual infectivity has been detected in multiple herpesviruses lacking NEC components (5, 18, 20). Our findings in MHV-68 align with previous transfection-based studies on NEC-knockout mutants (5), indicating that capsid escape can proceed without a fully functional NEC, albeit inefficiently and non-selectively, as evidenced by the increased levels of extracellular A- and B-capsids observed in our study.

The observation that capsids can escape the nucleus in the absence of a fully functional NEC raises the question of how such release is achieved. One potential route is NEBD. Although NEBD is most often associated with mitosis, Klupp et al*.* were the first to demonstrate that extensive passaging of their pseudorabies virus NEC mutants can induce NEBD as an alternative route for herpesvirus nuclear egress. They and colleagues have since elaborated this phenomenon in follow-up studies showing its modulation by cellular kinases such as CDKs and MEK1/2 through the regulatory function of the tegument protein pUL46 (18, 19, 41, 47). In their studies, NEBD became apparent upon extended passaging. Whether the low-level nuclear escape observed in unpassaged NEC mutants reflects the same underlying cellular process, a related phenomenon, or the interplay of multiple mechanisms remains unresolved. In our unpassaged MHV-68 mutant, EM revealed discontinuities in the nuclear envelope consistent with NEBD, suggesting that these events are not limited to viruses that have undergone extensive passaging. While these findings are compatible with NEBD contributing to capsid escape in our system, they do not exclude the possibility that additional processes may facilitate residual nuclear egress when the NEC is impaired.

The role of CDKs in herpesvirus replication is increasingly recognized, with activity linked to viral gene expression and egress (53, 54), and herpesvirus-encoded cyclins (e.g., MHV-68 v-cyclin) are known to associate with CDK2 and CDC2 (55). In our study, CDK inhibition reduced viral spread in both the parental virus and the ΔC-ORF69 mutant. Although CDKs are critical for viral replication, the mutant’s continued sensitivity suggests that this sporadic nuclear escape may also be supported by host factors regulated by cell-cycle kinases, such as those controlling nuclear envelope integrity. Conversely, the MEK1/2 inhibitor U0126 reduced plaque size in the parental virus, consistent with findings that viruses may modify MEK signaling to enhance spread (56), but did not affect ΔC-ORF69. This indicates that while MEK1/2 signaling optimizes efficient spread in the wild-type context, it is not required for the residual, non-selective spread observed in the ΔC-ORF69 context. While such host-mediated processes may permit limited capsid escape, they do not explain how capsid-type selectivity is normally imposed during nuclear egress.

The mechanism by which the NEC exerts selectivity for C-capsids remains a subject of debate. Current models propose selection based on the relative abundance of capsid-associated proteins, such as HSV-1 UL25 or its MHV-68/KSHV homolog ORF19. Since UL25 copy numbers are highest on C-capsids, followed by A- and B-capsids (3, 57), the NEC may favor capsids with high UL25 occupancy, providing a molecular basis for selectivity. However, this model presumes a highly accurate sensing mechanism that has not yet been proven. An alternative hypothesis posits that the asymmetric unit harboring the portal is the key determinant (58). The portal in C-capsids may possess unique features, such as a portal cap, distinct structural arrangement, or linkage to the viral genome, that are preferentially recognized by the NEC. While our study confirms the loss of capsid selectivity in the absence of a functional NEC, the data support a model in which the NEC lattice functions as a selective gate during nuclear egress. When this selective gate is absent, capsids are retained within the nucleus and only gain access to the cytoplasm at late stages of infection, coincident with loss of nuclear envelope integrity, resulting in the non-selective release of all capsid forms.

If capsid selectivity is imposed during nuclear egress, this raises the question of whether later stages of virion maturation also contribute to capsid quality control. Identifying primary envelopment as the capsid quality control step has implications for the selectivity of the later stages of capsid egress. This observation, in addition to the presented direct EM data, suggests that tegumentation and secondary envelopment will occur on A-, B-, and C-type capsids indiscriminately. Our observations further highlight a critical distinction between nuclear and cytoplasmic quality control mechanisms. The detection of enveloped A-, B-, and C-capsids in the supernatant and extracellular space of both ΔC-ORF69 and the parental virus, particularly late in infection, suggests that secondary envelopment does not impose a strict selectivity control step regarding capsid classification.

Together, these observations imply that nuclear capsid escape likely occurs during a temporal window where nuclear integrity is compromised, potentially through transient ruptures, while the cytoplasm remains sufficiently metabolically active to support tegumentation and envelopment. Together, these observations reinforce the NEC as the selective gate that normally restricts nuclear egress to C-capsids. In the ΔC-ORF69 mutant, this specific interaction is lost, and capsids are only released when the nuclear envelope is compromised.

This model raises the question of how individual NEC components, and ORF69 in particular, contribute to capsid docking and selective nuclear egress. Regarding the structural role of ORF69, homologs in HSV-1 and PrV (UL31) are proposed to guide capsids to the nuclear envelope and facilitate interactions with the inner nuclear membrane protein UL34 (59–64). This aligns with our observation that nuclear C-capsids in ΔC-ORF69 cells cluster near the nuclear envelope but do not interact with it. This suggests that while the residual N-terminus of ORF69 is predicted to interact with ORF67, it is also evidenced by the accumulation of NEC-associated vesicular structures in the cytoplasm or perinuclear space, similar to what is observed in ORF69 and ORF67 and homologs co-expression (5, 42, 43), it is insufficient to mediate stable capsid docking. Notably, the N-termini of MHV-68 ORF69 and its homologs are highly variable and have been implicated in diverse functions (65). In HSV-2, UL31 is associated with DNA replication, packaging, and the DNA damage response (28), whereas in HSV-1, it plays a role in membrane budding (66). We did not observe a packaging defect comparable to that observed in the homologous γ-herpesvirus EBV mutants (40), suggesting that the residual N-terminal region of ORF69 may contribute to packaging efficiency, though further systematic investigation is required to confirm this.

In conclusion, our findings contribute to growing evidence that herpesviruses utilize both NEC-dependent and alternative nuclear egress routes, with the NEC acting as the dominant mechanism for efficiency and the essential checkpoint for capsid selection. The observation that secondary envelopment permits the exit of defective capsids further reinforces the NEC as the final major quality-control step in the replication cycle. While alternative egress routes, likely involving host-mediated nuclear envelope compromise, offer a viable but highly inefficient survival option, the reduced fitness of ΔC-ORF69 emphasizes the critical importance of NEC-mediated selection in optimizing viral propagation.

## MATERIALS AND METHODS

### Structure prediction

For the structure prediction of NEC heterodimers and heterooligomers of MHV-68, EBV, and KSHV, protein sequences were downloaded from https://www.uniprot.org using the entry numbers listed in the table of our HerpesFolds database (67). AlphaFold 3 (32) predictions were performed through the AlphaFold Server at https://alphafoldserver.com. UCSF ChimeraX version 1.8 (https://www.cgl.ucsf.edu/chimerax) was used to visualize structures. Data analysis was carried out with custom-written Python scripts, generated in part with the assistance of large language models and thoroughly tested manually, as previously published (67).

### Viruses

The virus mutants used in this study are based on the BAC backbone previously described (68, 69). MHV-68 ORF65-mCherry, referred to as the parental virus throughout this study, was generated and characterized as described previously (70). To insert the expression cassettes encoded by the above-described rescue plasmids into the MHV-68 BAC, we used the single-step FRT/Flp system originally established for MCMV (9). For this purpose, *Escherichia coli* strain DH10B (Invitrogen), containing the MHV-68 BAC backbone and the temperature-sensitive Flp recombinase expression plasmid pCP20 (71), was transformed with various R6K-γ-driven pOTO constructs (9) carrying ORF65-fusion cassettes. Transformation and selection were performed as previously described (72). The ΔC-ORF69 BAC was generated using a two-step replacement procedure, as described (73), with the shuttle plasmid outlined above. Correct recombination was verified by analysis of the restriction patterns of the respective ΔC-ORF69 and the parental virus BAC by restriction digest with EcoRI, HindIII, and SacI for 1 h at 37°C and gel electrophoresis on a 0.8% agarose gel run at 180 V for 2 h and subsequently 30 V overnight.

### Cells

The experiments were carried out on the NIH3T3 mouse fibroblast cell line. For trans-complementing ORF69, NIH3T3 cells were transduced using a third-generation lentiviral system. To generate a lentiviral vector using the Gateway Recombination Cloning Technology, ORF69 was amplified with flanking attB1 and attB2 sites. It was cloned into pDONR221 using a BP reaction according to the manufacturer’s protocol (Thermo Fisher, USA), yielding the entry clone. An LR reaction was then performed between the entry clone and the destination vector pLenti CMV Puro Dest (pLenti CMV Puro DEST (w118-1) was a gift from Eric Campeau & Paul Kaufman) (Addgene plasmid #17452) to generate the pLenti_CMVPuro_ORF69 construct. Lentiviral particles were produced by transfecting human 293XT cells with pLenti_CMVPuro_ORF69 and the necessary packaging plasmids. Virus-containing supernatants were harvested and used to transduce NIH3T3 cells. Transduced cells were subjected to puromycin selection to enrich for stable ORF69 expression. These cells were subsequently used to produce virus stocks of ΔC-ORF69 under non-selective conditions.

Primer sequences were as follows: attB1_ORF69 forward, 5′-GGGGACAAGTTTGTACAAAAAAGCAGGCTTAatgcgctcaacaggctctg-3′; attB2_ORF69 reverse, 5′-GGGGACCACTTTGTACAAGAAAGCTGGGTTttgctgagaaagacgagatacaatgttga-3′.

### Plaque size measurement

NIH3T3 or NIH3T3ORF69 cells were seeded on 6-well plates at a density of 5.4 × 10^5^ cells per well to achieve 80% confluency the following day. Cells were infected with serial dilutions (On NIH3T3: 10^−3^ for the parental virus, 10^−1^ for ΔC-ORF69, and on NIH3T3ORF69: 10^−1^ for ΔC-ORF69) with either the parental virus or ΔC-ORF69. At 2 h post-infection (hpi), the medium was replaced with 2 mL DMEM supplemented with 2% FCS and 0.6% methylcellulose. At 4 dpi, cells were fixed in 4% PFA for 20 min at RT and stored in PBS at 4°C until further analysis. Whole wells were imaged on a Leica DMi8 inverse widefield microscope with a THUNDER unit (Leica) and Leica HCX PL FLUOTAR 10x/0.3 NA objective. The setup also included an LED-based illumination system with excitation at 395 nm, 440 nm, 510 nm, 550 nm, and 640 nm, and respective filter sets. For analysis, 49 × 49 FOV (12,864 px × 12,864 px) with a 10% overlap between FOVs was processed with a custom image processing pipeline in Fiji ImageJ (v.1.54f). A Gaussian blur (“Σ = 10”) was applied to remove noise and improve segmentation, followed by manually removing air bubbles or dust. The threshold “Otsu” was used and adjusted according to the cellular background signal to create a binary mask, which was then processed using “Fill Holes.” The lower size threshold for particle analysis was determined by measuring the mean size of single infected cells and comparing the mean size to infected cell clusters of two to three cells. Areas with a size range of >1,000 µm² were defined as a plaque, a cell cluster of two to three cells. Results were plotted using GraphPad Prism (v.10.4.1). The three groups were tested for normality, and the Kruskal-Wallis test was used to compare the groups. For visualization of the imaging pipeline, the binary masks were color-coded using the “BAR” plugin with the “mpl-magma” LUT (range: 0–20,000 µm²) in Fiji ImageJ (v.1.54f).

### Passaging virus, preparation of viral DNA, and sequencing via Illumina sequencing

To assess the genomic effects of MHV-68 ΔC-ORF69 passaging on non-complementing cells, 2 × 10⁶ NIH3T3 cells were seeded in a 10 cm tissue culture dish one day before infection. Cells were infected with 1,000 PFU of ΔC-ORF69 and incubated at 37°C with 5% CO_2_ until full CPE was observed. The virus was harvested by scraping and resuspending cells in medium, then stored at −80°C for one freeze-thaw cycle, generating passage 1 (Pass 1). For subsequent passages, fresh NIH3T3 cells were seeded and infected with the same virus volume as the prior passage. This procedure was repeated until passage 9 (Pass 9). For passage 10 (Pass 10), the supernatant was collected, centrifuged at 200 rcf for 3 min to pellet debris, filtered (0.45 µm), and concentrated via centrifugation at 16,000 rcf for 2 h at 4°C. The upper supernatant was discarded, leaving 2 mL of concentrated virus. For DNA extraction, 1 mL of the virus suspension was incubated for 1 h at 50°C with 50 µg Proteinase K, 1% SDS, and 1 µL RNase A. The sample was processed using phase-lock tubes pre-spun at 20,000 rcf for 2 min at 4°C. Sequential extractions with phenol-chloroform-isoamyl alcohol were performed, followed by chloroform extraction. The aqueous phase was pooled, and DNA was precipitated with sodium acetate (3 M, 1/10 volume) and isopropanol (4/5 volume), incubated at RT for 25 min, and centrifuged at 18,000 rcf for 30 min at 4°C. The DNA pellet was washed with 70% ethanol, and the pellet was dissolved in distilled water overnight at 4°C. ΔC-ORF69 Pass 10 DNA was sequenced using an Illumina MiSeq platform. Library preparation was performed using the NEBNext Ultra II FS DNA Library Prep Kit for Illumina (NEB, E7805), followed by paired-end sequencing (2 × 151  bp) on an Illumina MiSeq platform, generating approximately 1 million read pairs. Reference-guided *de novo* assembly with paired-end reads was performed using SPAdes genome assembler (74 ) with the parameters “--careful” and “--only-assembler,” using an *in silico* MHV-68 ΔC-ORF69 genome as reference. Variant calling was performed using GATK HaplotypeCaller with the option “!--dont-use-soft-clipped-bases.” Variant filtration was performed using the parameters “QD < 2.0,” “FS > 60.0,” “MQ < 30.0,” “MQRankSum < –12.5,” and “ReadPosRankSum < –8.0” (75).

### Sanger sequencing

The ORFs were amplified directly from purified viral DNA (BAC, Pass 1, Pass 5, Pass 10) as a template. Reactions were performed using the KOD Xtreme HotStart Polymerase (Sigma Aldrich, USA) according to the manufacturer’s instructions and primers listed below. PCR products were separated by 1% agarose gel electrophoresis and purified using the NucleoSpin Gel and PCR Clean-up Kit (Macherey-Nagel, Germany) according to the manufacturer’s protocol. The purified PCR fragments were sent for Sanger sequencing (ORF67, Microsynth Seqlab GmbH, Germany; ORF69, Eurofins Genomics, Germany), sequencing histograms were analyzed and aligned to the reference sequence using SnapGene (GSL Biotech LLC, USA).

Primer sequences were as follows: ORF69 forward (F), 5′-ATGCGCTCAACAGGCTCTGCT-3′; ORF69 reverse (R): 5′-TTGCTGAGAAAGACGAGATACAATGTTGAAG-3′; ORF67 F, 5′-CAGGGATACCATATTGACCCTGGTGGAC-3′; ORF67 R, 5′-GTCCAAGGCCCCCATCACCATAC-3′; ORF4 F, 5′-GTGGCCCTACCCCGAATCTC-3′; ORF4 R, 5′-GTAACCACCCACGCCGAG-3′.

### Preparation of samples for TEM

For TEM sample preparation, NIH3T3 cells were seeded in µ-Dish 35 mm, high Grid-500 (Ibidi, Germany) and infected with an MOI of 0.1 with either the parental virus or ΔC-ORF69. After either 1 dpi (parental virus) or 4 dpi (ΔC-ORF69), cells were fixed with 2% PFA and 2.5% GA in PBS for 20 min at RT, followed by post-fixation in 2.5% GA in PBS for 1 h at 4°C. For the late time point in ΔC-ORF69 (21 dpi), after formation of a visible plaque, cells were fixed with 2% PFA and 2.5% GA in PBS for 20 min at RT, followed by post-fixation in 2.5% GA in PBS for 14 days at 4°C. The position of infected cells was selected using a Nikon Ti2 spinning-disk fluorescence microscope equipped with a Yokogawa CSU-W1 SoRa spinning disc unit, two Hamamatsu Orca Fusion BT sCMOS cameras, and a 20x PLAN APOλD NA = 0.80 (Nikon). The setup also included 405, 445, 488, 515, 561, and 638 nm laser lines and respective filter sets. After the localization of cells of interest, samples were washed with PBS and incubated in 1% osmium in PBS for 20 min, then stained with uranyl acetate for 20 min in the dark. After washing, samples were dehydrated in increasing ethanol concentrations (50%, 70%, 90%, and 100%) for 10 min each. For embedding in epoxy resin (EPON) (76), samples were incubated in 50% EPON for 30 min, 70% EPON for 1.5 h, and 100% EPON overnight. After the 100% EPON was renewed twice, samples were polymerized at 60°C overnight. Ultra-thin sections (50 nm) of multiple cells and depths were cut with a diamond knife and transferred onto copper grids. The grids were analyzed via TEM using a FEI Tecnai G20 (Thermo Fisher, USA).

### Embedding virus pellets from supernatant for TEM

For supernatant preparation for TEM, 5 × 10⁶ NIH3T3 cells were seeded on two 15 cm dishes and infected the following day with a 0.1 MOI of ΔC-ORF69 or the parental virus. After 1 h, the medium was replaced with prewarmed DMEM containing 5% FCS. The supernatant was collected at 3 dpi for ΔC-ORF69 or 4 dpi for the parental virus, centrifuged at 2,300 rcf for 5 min, and the cleared supernatant was transferred to a new tube. The supernatant was centrifuged again, and 3 mL of 30% sucrose cushion was then centrifuged at 15,000 rcf for 1.5 h at 4°C. After removing the supernatant, the virus pellet was resuspended in 1 mL PBS with 1% FBS and centrifuged at 15,000 rcf for 1.5 h at 4°C. The virus pellet was fixed in 4% PFA in PBS and, depending on the consistency of the pellet, either directly processed for TEM or transferred into capillary tubes for support (hollow cellulose fiber; type LD OC O2; Microdyn Wuppertal, Germany) (45). The tubes were sealed with a ring curette, processed according to the TEM preparation of cells, and analyzed by TEM using a FEI Tecnai G20 (Thermo Fisher, USA).

### Embedding late-stage infected cell pellets for TEM

For preparation of infected cell pellets for TEM, 5 × 10⁶ NIH3T3 cells were seeded on two 15 cm dishes and infected the following day with a 0.1 MOI of either ΔC-ORF69 or the parental virus. After 1 h, the medium was replaced with prewarmed DMEM containing 5% FCS. At 4 dpi, the supernatant was collected to recover already detached, rounded-up cells. The infected cell monolayer was gently rinsed with 10 mL PBS to detach additional rounded-up cells, and the PBS rinse was pooled with the supernatant. The pooled cell suspension was centrifuged at 2,300 rcf for 5 min. The supernatant was aspirated, and the resulting cell pellet was processed for TEM using the same workflow described above for the virus pellet.

### Quantification of capsids

Fluorescently labeled infected cells were initially identified in fluorescence microscopy images before correlating them with their corresponding regions in EM images. Individual EM images were matched to specific cells, and each image’s depth within the cell was categorized by its grid number. Capsids in each image were manually scored using the “Point Tool” in Fiji/ImageJ (v.1.54f), with distinct counters assigned to different capsid types: Counter 0 = A-capsid, Counter 1 = B-capsid, Counter 2 = C-capsid (mature capsid), and Counter 3 = unclear (not classifiable).

For calculating the proportion of enveloped versus unenveloped capsid types, the data were re-scored with additional counters. For the parental virus, unenveloped capsids were re-scored because they represented the minor population, whereas for ΔC-ORF69, enveloped capsids were re-scored, as they represented the minor population. Counters were defined as follows: Counter 4 = A-capsid, Counter 5 = B-capsid, Counter 6 = C-capsid.

The recorded counts were documented in a Microsoft Excel sheet, including metadata such as cell identifier, grid number, image number, subcellular localization (cytoplasm, nucleus, extracellular capsids, or supernatant), and the respective capsid counts. This structured data set ensured traceability back to the individual images. Total capsid numbers and ratios were calculated using Microsoft Excel, and data visualization was performed using GraphPad Prism (v.10.4.1).

### Live-cell imaging

NIH3T3 cells were seeded in Ibidi 8-well glass-bottom slides (Ibidi, Germany) at a density of 1.2 × 10⁴ cells per well. Cells were infected with an MOI of 0.1 per well with either the parental virus or ΔC-ORF69. At 2 hpi, the medium was replaced with 200 µL per well DMEM supplemented with 2% FCS. At 1 dpi, the nuclei were counterstained with 10 µg/mL Hoechst 33342 in PBS for 15 min at 37°C. The cells were continuously imaged for 40 s on a Nikon Ti2 spinning disk fluorescence microscope equipped with a Yokogawa CSU-W1 SoRa spinning disc unit, two Hamamatsu Orca Fusion BT sCMOS cameras, and a 100× Apo TIRF NA = 1.49 objective (Nikon). The setup also included 405, 445, 488, 515, 561, and 638 nm laser lines and respective filter sets. An environmental control system kept physiological growth conditions constant at 37°C, with 5% CO_2_. A representative image of the cells was color-coded based on intensity using the LUT “mpl-magma” in Fiji ImageJ (v.1.54f).

### Analyzing cytotoxicity and optimal inhibitor concentration on NIH3T3 cells

NIH3T3 cells were seeded in 24-well plates at a density of 1.08 × 10⁵ cells per well for high concentrations (10 µM–100 µM) or 1.11 × 10⁵ cells per well for low concentrations (0.1 µM–10 µM) in 500 µL DMEM supplemented with 10% FCS and incubated overnight at 37°C, 5% CO_2_. U0126 (art. no: 662005, Sigma Aldrich, USA) and Roscovitine (art. no: R772, Sigma Aldrich, USA) were prepared as 10 mM stock solutions in DMSO, aliquoted, and stored at −20°C. The following day, the medium was replaced with 500 µL per well of DMEM containing 2% FCS and 0.6% methylcellulose, supplemented with U0126 (10, 25, 50, or 100 µM), Roscovitine (0.1, 0.5, 1, 2.5, 5, 10, 25, 50, or 100 µM), DMSO (≤1%, vehicle control), or PBS (negative control), and incubated protected from light. The overlay was renewed daily with an overlay containing the freshly diluted respective inhibitor concentrations or control solutions. At 3 dpi, cytotoxic effects were assessed by aspirating the overlay, washing cells with 500 µL PBS, and detaching them with 200 µL TrypLE Express Enzyme (1×) (Gibco, Thermo Fisher Scientific, USA). Trypsinization was blocked with 800 µL DMEM containing FCS, and cells were resuspended and transferred to Eppendorf tubes for counting to determine cell numbers. Values were plotted using GraphPad Prism (v.10.4.1).

### Inhibitor studies

NIH3T3 cells were seeded in Ibidi 8-well glass-bottom slides (Ibidi, Germany) at a density of 6.0 × 10⁴ cells per well for inhibitor treatment conditions and 4.5 × 10⁴ cells per well for control conditions to achieve approximately 90% and 70% confluency, respectively, the following day. Cells were infected with ~60 PFU per well with either the parental virus or ΔC-ORF69. At 2 hpi, the medium was replaced with 200 µL per well DMEM supplemented with 2% FCS and 0.6% methylcellulose, and either PBS (0.5%), DMSO (0.5%), Roscovitine (5 µM), or U0126 (50 µM). The overlay was renewed daily by removing as much of the existing overlay as possible and replacing it with fresh overlay containing controls or inhibitors. At 3 dpi, cells were fixed in 4% PFA for 20 min at RT and stored in PBS at 4°C until further analysis. Cells were imaged with a 20 × 20 field of view with an ROI size of 1,024 × 1,024 pixels with 15% overlap and stitching via blending on a Nikon Ti2 (Nikon) spinning-disk fluorescence microscope equipped with a Yokogawa CSU-W1 SoRa spinning disc unit, two Hamamatsu Orca Fusion BT sCMOS cameras, and a 10× PLAN APOλD NA = 0.45 objective (Nikon). The setup also included 405, 445, 488, 515, 561, and 638 nm laser lines and respective filter sets. FOVs were analyzed with a custom image processing pipeline in Fiji ImageJ (v.1.54f). A Gaussian blur (“Σ = 15”) was applied to remove noise and improve segmentation, followed by thresholding using “Otsu” ("110-max") to generate a binary mask. The mask was processed with “Fill Holes” to ensure continuous regions. Plaques, as defined for plaque size measurement, were identified using “Analyze Particles” with a size threshold of >1,000 µm² for ΔC-ORF69, as defined in the method for the plaque size measurement, and >3,000 µm² for the parental virus, to exclude small plaques caused by reinfection events due to the daily change of the overlay. Results were plotted using GraphPad Prism (v.10.4.1), the groups were tested for normality, and the Kruskal-Wallis test was used to compare multiple groups.

## Data Availability

All data sets generated and analyzed during this study are publicly available via the Zenodo repository. The data set includes microscopy data, comprising files from electron microscopy, live-cell imaging, and conventional light microscopy, as well as the gel image of the BAC restriction digest. Sequencing data, including next-generation sequencing (NGS) and Sanger sequencing experiments, were also included. Protein structure predictions were generated using AlphaFold. The sequences of ΔC-ORF69 and ΔC-ORF69 Pass 10 have been deposited in the European Nucleotide Archive (ENA) (https://www.ebi.ac.uk/ena/browser/view/PRJEB108604) under accession number PRJEB108604. The complete data set can be accessed via Zenodo (https://zenodo.org/records/15147695).
